# Supplementary material for: Mapping the drivers of within-host pathogen evolution using massive data sets
Source: Nat Commun. 2019 Jul 9;10:3017. doi: 10.1038/s41467-019-10724-w (PMC6616926; doi:10.1038/s41467-019-10724-w)
Supplement: Supplementary file 1 — Supplementary Information [file 41467_2019_10724_MOESM1_ESM.pdf]

Mapping the drivers of within-host pathogen evolution using massive data sets: Supplementary Information.

Palmer *et al.*[*]

[*]duncan.stuart.palmer@gmail.com

Supplementary Methods

Methods overview

We perform inference via Markov chain Monte-Carlo (MCMC) using the Li and Stephens approximation to the coalescent with recombination [1], with one further key approximation. Consider two data sets:

1. $\mathbf{D}$ = viral sequences for which host HLA data is also available.
2. $\mathbf{D}_\mathrm{B}$ = viral sequences for which host HLA data is not available.

As $\mathbf{D}_\mathrm{B}$ is very large, we can assume that $\mathbf{D}_\mathrm{B}$ represents the collection of viral sequences (subject to recombination) that a host can be infected with. A large number of sequences in $\mathbf{D}_\mathrm{B}$ results in short coalescence times between any given member of $\mathbf{D}$ and $\mathbf{D}_\mathrm{B}$, increasing our power to estimate parameters. By making this approximation, we may then assume that selection along the lineage joining a given member d of $\mathbf{D}$ to its nearest neighbour in $\mathbf{D}_\mathrm{B}$ occurred within the individual from whom the virus d was sampled. We have associated host HLA profiles for $\mathbf{D}$, and so may then estimate HLA-associated selection conditional on this HLA information. A cartoon of this model is shown in Figure 1*b*.

The Li and Stephens approximation allows us to evaluate the probability that a member of $\mathbf{D}$ arises as an imperfect mosaic of sequences in $\mathbf{D}_\mathrm{B}$ using a hidden Markov model (HMM). To model this process, we must specify a recombination model, and model of sequence evolution. We assume that recombination is site dependent, and occurs with probability r_i between site $i-1$ and i to any other member of $\mathbf{D}_\mathrm{B}$ uniformly at random. We model sequence change using the NY98 codon model [2] (which distinguishes transitions from transversions via the transition/transversion ratio; κ, and non-synonymous changes from synonymous changes via the $\frac{dN}{dS}$ ratio; ω), with some modifications. To incorporate HLA-associated selection for escape, we define a consensus non-escaped strain $\mathscr{C}$ for the region to be analysed. A scaling, $\prod_{h\in\mathbf{H}}\gamma_{h,i}$, dependent on the host's HLA profile, $\mathbf{H}$, then modifies any non-synonymous codon change from this consensus strain. We model reversion in a similar manner: a boost in codon substitution rate towards the consensus codon $\mathscr{C}$ at each site, ζ_i. Note that reversion only acts on non-synonymous changes toward the consensus codon $\mathscr{C}$, and as such the parameterisation is identifiable. To switch from rates to probabilities we integrate over the length of a lineage connecting a member of $\mathbf{D}$ to $\mathbf{D}_\mathrm{B}$ assuming a standard coalescent. For example for a non-synonymous transition from $\mathscr{C}_i$ to some codon C,

$$P\left(\mathscr{C}_i \to C|\, \mathbf{H}, \mathbf{\Theta}\right) \approx \left(\phi \frac{a_i \prod_{h\in\mathbf{H}}\gamma_{h,i}}{A} + (1-\phi)\,\pi_C\right)\left(1 - \frac{k}{k+A}\right) \tag{1}$$

where ϕ is the empirically estimated probability of observing a single codon change along the lineage joining $\mathbf{D}$ to $\mathbf{D}_\mathrm{B}$, A is the total codon substitution rate out of $\mathscr{C}_i$, a_i is the number of non-synonymous transitions from $\mathscr{C}_i$, k is the number of sequences in $\mathbf{D}_\mathrm{B}$ and π_C is the empirical probability of observing C given a ≥ 2 step codon change. We approximate the probability in this manner to avoid computationally expensive matrix exponentiation. Given recombination probabilities and codon transition probabilities for all possible codon changes and the Li and Stephens approximation, we are armed with an HMM to describe an approximation to the process generating members of $\mathbf{D}$. We can therefore use the forwards and backwards algorithms to integrate over all paths through $\mathbf{D}_\mathrm{B}$ to generate each member of $\mathbf{D}$ in turn. We then take the product over all members of $\mathbf{D}$ to approximate the likelihood:

$$P\left(\mathbf{D}|\mathbf{D}_\mathrm{B}, \mathcal{H}, \mathbf{\Theta}\right) \approx \prod_{D_j\in\mathbf{D}} P\left(D_j|\mathbf{D}_\mathrm{B}, \mathbf{H}_j, \mathbf{\Theta}\right) \tag{2}$$

where $\mathcal{H}$ denotes HLA information (across members of $\mathbf{D}$ and $\mathbf{D}_\mathrm{B}$), $\mathbf{H}_j$ is the collection of HLA types of the host associated to viral sample $D_j \in \mathbf{D}$, and $\hat{\pi}$ is the function describing the Li and Stephens approximation with our model of recombination and codon evolution.

Evaluating $\prod_{D_j \in \mathbf{D}} \hat{\pi}\left(D_j|\mathbf{D}_\mathrm{B}, \mathbf{H}_j, \mathbf{\Theta}\right)$ in practice becomes computationally difficult as $\mathbf{D}_\mathrm{B}$ increases in size, as it involves the evaluation of arrays of dimension $|\mathbf{D}_\mathrm{B}| \times |\mathbf{D}| \times |\text{codon sequence}|$. To increase computational efficiency, we restrict $\mathbf{D}_\mathrm{B}$ to a subset $\mathbf{D}_{\mathrm{B}_j}$ for each member of $\mathbf{D}$. In our simulations and analyses we use a simple Hamming distance restriction: the closest n sequences by Hamming distance to define each $\mathbf{D}_{\mathrm{B}_j}$.

Now that we are able to evaluate approximate likelihoods rapidly, we can perform MCMC to obtain posterior distributions for our parameters of interest:

- $\frac{dN}{dS}$ at each site, ω_i.
- HLA-associated selection at each site, $\gamma_{h,i}$.
- Recombination probabilities between neighbouring sites, r_i.
- Synonymous transversion rate, μ.

The Li and Stephens approximation and the forwards-backwards algorithms

We use the Li and Stephens [1] approximation, as it captures many of the properties of the coalescent with recombination in a computationally efficient manner. We describe the approximation and explain our modifications.

Let $\mathbf{\Theta}$ denote a collection of parameters governing codon substitution and let $\mathbf{D} = \{D_1, D_2, \ldots, D_n\}$ denote a collection of sequences from n samples. The approximation follows from observing that the likelihood $P(D_1, D_2, \ldots, D_n|\mathbf{\Theta})$ may be written as a product of conditional likelihoods, each of which is then approximated by a function which we denote $\hat{\pi}$. I.e.:

$$P(D_1, D_2, \ldots, D_n|\mathbf{\Theta}) = P(D_1|\mathbf{\Theta})P(D_2|D_1, \mathbf{\Theta}) \ldots P(D_n|D_1, D_2, \ldots, D_{n-1}, \mathbf{\Theta}) \quad (3)$$
$$\approx \hat{\pi}(D_1|\mathbf{\Theta})\hat{\pi}(D_2|D_1, \mathbf{\Theta}) \ldots \hat{\pi}(D_n|D_1, D_2, \ldots, D_{n-1}, \mathbf{\Theta}). \quad (4)$$

The right hand side of (4) is known as the product of approximate conditionals (PAC) likelihood as each conditional likelihood $P(D_{k+1}|D_1, D_2, \ldots, D_k, \mathbf{\Theta})$ is approximated using the function $\hat{\pi}$. Li and Stephens [1] use a hidden Markov model (HMM) for $\hat{\pi}$ that captures a collection of desirable properties of the true likelihood function, but is computationally tractable, because that the $(k+1)^{\text{th}}$ sequence is an imperfect mosaic of the first k sequences due to mutation and recombination. For the $(k+1)^{\text{th}}$ sequence, the hidden states are the first k sequences. We denote $X_1, X_2, \ldots, X_m$ as the hidden Markov chain that emits the sequence D_{k+1}, where X_j is the sequence being copied from (SCF) at position j and m is the length of the sequence. Between each site, the SCF switches to a new sequence l with probability $q_{x,i}(j)$ (which may depend on the current SCF; x, the new SCF; i, and the current site; j). Given a hidden state at position j, the probability of observing the codon in the observed sequence D_{k+1} is generated by a model of codon substitution. To generate $\hat{\pi}(D_{k+1}|D_1, D_2, \ldots, D_k, \mathbf{\Theta})$ we integrate over all possible paths through sequences $D_1, D_2, \ldots, D_k$ that can generate the sequence D_{k+1} through a combination of jumping between these k sequences (recombination) and error in copying (mutation), as illustrated in Figure 1*b*.

As the approximation to the process generating sequence D_k is an HMM, we can appeal to the forwards and backwards algorithms [3] to evaluate $\hat{\pi}(D_{k+1}|D_1, D_2, \ldots, D_k, \mathbf{\Theta})$ for each k. The forwards algorithm proceeds as follows:

Let $D_{k+1}[1:j]$ denote the codons at the first j sites of sequence $k+1$ and let $f_{i,j}$ denote the probability of observing all codons in D_{k+1} up to site $j-1$ and copying the codon in position j from sequence i. Then $f_{i,j} = P(D_{k+1}[1:j], X_j = i|D_1, D_2, \ldots, D_k, \mathbf{\Theta})$. Let the probability of copying the codon at

position j from sequence i be denoted $\varepsilon_{i,j}$. This is the probability of an observed state given a hidden state, and is known as an emission probability in the language of HMMs. Then $f_{i,1}$ is just an emission probability and easily computed if we have a uniform prior on the SCF at the first position. We then calculate $f_{i,j}$ for $j \geq 2$ recursively as follows:

$$f_{i,j} = \varepsilon_{i,j} \sum_{x=1}^{k} f_{x,j-1} q_{x,i}(j), \tag{5}$$

where, as defined earlier, $q_{x,i}(j)$ is the combined probability of recombination and the destination sequence being sequence i. Note that we make the implicit assumption that recombination events may only occur between codons and not within them. If we assume that all sequences are equally likely to recombine (as in [1] and [4]) then (5) may be simplified to

$$f_{i,j} = \varepsilon_{i,j} \left((1 - r_j) f_{i,j-1} + \frac{r_j}{k} \sum_{x=1}^{k} f_{x,j-1} \right), \tag{6}$$

where r_j is the probability of recombination between the $(j-1)^{\text{th}}$ and j^{th} position.

For the $(k+1)^{\text{th}}$ sequence,

$$\hat{\pi}(D_{k+1} | D_1, D_2, \ldots, D_k, \mathbf{\Theta}) = \sum_{x=1}^{k} f_{x,m}. \tag{7}$$

We may similarly run the backwards algorithm, starting at the last site and evaluating iteratively towards the first site. Let $\mathbf{D}_1^k = \{D_1, D_2, \ldots, D_k\}$. $b_{i,j}$ is constructed such that

$$f_{i,j} b_{i,j} = P(X_j = i, D_{k+1} | \mathbf{D}_1^k, \mathbf{\Theta}) \tag{8}$$

$$\Rightarrow \quad P(D_{k+1}[1:j], X_j = i | \mathbf{D}_1^k, \mathbf{\Theta}) b_{i,j} = P(X_j = i, D_{k+1} | \mathbf{D}_1^k, \mathbf{\Theta}) \tag{9}$$

$$\Rightarrow \quad b_{i,j} = P(D_{k+1}[j+1:m] | X_j = i, D_{k+1}[1:j], \mathbf{D}_1^k, \mathbf{\Theta}) \tag{10}$$

$$\Rightarrow \quad b_{i,j} = P(D_{k+1}[j+1:m] | X_j = i, \mathbf{D}_1^k, \mathbf{\Theta}) \tag{11}$$

where the last implication is a consequence of the Markov property. The $b_{i,j}$s for the $(k+1)^{\text{th}}$ sequence can then be generated iteratively as follows

$$b_{i,m} = 1 \tag{12}$$

$$b_{i,j} = \sum_{x=1}^{k} b_{x,j+1} \varepsilon_{x,j+1} q_{x,i}(j) \tag{13}$$

$$b_{i,j} = \varepsilon_{i,j+1} (1 - r_{j+1}) b_{i,j+1} + \frac{r_{j+1}}{k} \sum_{x=1}^{k} b_{x,j+1} \varepsilon_{x,j+1}. \tag{14}$$

Hence we can calculate

$$\sum_{i=1}^{k} f_{i,j} b_{i,j} = \sum_{i=1}^{k} P(X_j = i, D_{k+1} | \mathbf{D}_1^k, \mathbf{\Theta}) \tag{15}$$

$$= \hat{\pi}(D_{k+1} | \mathbf{D}_1^k, \mathbf{\Theta}). \tag{16}$$

Note that the approximation of $P(D_1, D_2, \ldots, D_n | \mathbf{\Theta})$ using this HMM is dependent upon the ordering of $\{D_1, D_2, \ldots, D_k\}$. This is often resolved by randomly permuting the ordering some fixed number of times and determining an average [4], or by letting the order be an unknown parameter [5]. Here we remove the ordering issue by considering each query sample independently.

In our inference problem we have two data sets. One query data set; $\mathbf{D}$ for which we have associated host HLA information and another larger reference data set; $\mathbf{D}_\text{B}$ without host HLA information. Recall from our model summary that we make two important assumptions:

1. All selection along the lineages connecting a member of $\mathbf{D}$ to $\mathbf{D}_\mathrm{B}$ occurred within the host of the member of $\mathbf{D}$.
2. The reference data set $\mathbf{D}_\mathrm{B}$ is a good approximation of the distribution of viruses an individual can be infected with (which is reasonable when $|\mathbf{D}_\mathrm{B}| \gg 0$).

These two assumptions allow us to apply the Li and Stephens approximation. In the HMM we require emission probabilities. For our model these will be probabilities of codon substitutions in the presence of some HLA profile. Making assumption 1 allows us to approximate $P(\mathbf{D}|\mathbf{D}_\mathrm{B}, \mathbf{\Theta})$. This is because we have HLA genotype information for the members of $\mathbf{D} = \{D_1, D_2, \ldots, D_n\}$ which we can use to determine the relevant emission probabilities. I.e. The first assumption allows us to use the sequence data in $\mathbf{D}_\mathrm{B}$:

$$P(\mathbf{D}|\mathbf{D}_\mathrm{B}, \mathbf{\Theta}) = P(D_1|\mathbf{D}_B, \mathbf{\Theta})P(D_2|\mathbf{D}_B, D_1, \mathbf{\Theta}) \ldots P(D_n|\mathbf{D}_B, D_1, D_2 \ldots, D_{n-1}, \mathbf{\Theta}). \tag{17}$$

Using assumption 2, we may write

$$\begin{aligned} P(\mathbf{D}|\mathbf{D}_\mathrm{B}, \mathbf{\Theta}) &\approx P(D_1|\mathbf{D}_B, \mathbf{\Theta})P(D_2|\mathbf{D}_B, \mathbf{\Theta}) \ldots P(D_n|\mathbf{D}_B, \mathbf{\Theta}) & (18)\\ &\approx \hat{\pi}(D_1|\mathbf{D}_B, \mathbf{\Theta})\hat{\pi}(D_2|\mathbf{D}_B, \mathbf{\Theta}) \ldots \hat{\pi}(D_n|\mathbf{D}_B, \mathbf{\Theta}). & (19)\end{aligned}$$

In other words, assumption 2 results in the approximation that $\mathbf{D}$ is generated by independent realisations of recombination and mutation through $\mathbf{D}_\mathrm{B}$. This avoids any need for averaging over orderings of $\mathbf{D}$, allowing rapid evaluation of the approximate likelihood.

MCMC moves

Within the MCMC, we have three main classes of move. Moves to alter a parameter at a single site or window of sites, moves to merge or split windows, and moves to expand and contract selection windows to push them around the genome. When adding HLA-associated selection to our codon model of substitution, we considered separate parameters to scale selection away from consensus in the presence of each host HLA type at each site ($\gamma_{h,i}$), and parameters to model reversion towards consensus at each site (ζ_i). In order to increase speed we alter these parameters within selection windows in our MCMC scheme. The selection windows that we discuss are analogous to the selection windows and recombination windows described in [4]. We now introduce some notation:

Let $\mathcal{S} := \{s_1, s_2, \ldots, s_S\}$ be a collection of sites that split windows, and let $|\mathcal{S}| = S$. We may then alter parameters occurring within selection windows $[s_k, s_{k+1})$, $s_k \in \mathcal{S} \cup \{1, l\}$ (defining $s_0 = 1,\ s_{S+1} = l$). The number of selection windows is $S + 1$, and parameters are constant across these windows.

For recombination and selection, the parameters r_i and ω_i are estimated between each pair of neighbouring sites $(i-1, i)$, and at each site i respectively. For HLA associated selection and reversion parameter changes, moves are applied to a window of these parameters. Note, for each HLA type h we assign a distinct collection of selection windows.

Selection parameters ω_i. We only use a single type of MCMC move to alter ω_i across the codon sequence: changing ω_i at a given site i. We assume an exponential prior with parameter λ_ω for ω_i. Following the existing model [4], a new value ω_i' is chosen such that $\omega_i' = \omega_i \exp(U)$, where $U \sim \mathrm{Uniform}(-1, 1)$ and the acceptance probability is

$$\alpha_\omega(\mathbf{\Theta} \to \mathbf{\Theta}') = \min\left\{1, \frac{L(\mathbf{\Theta}'|\mathbf{D})}{L(\mathbf{\Theta}|\mathbf{D})}\frac{P(\omega_i')}{P(\omega_i)}\frac{q(\omega_i' \to \omega_i)}{q(\omega_i \to \omega_i')}\right\} \tag{20}$$

$$\Rightarrow \alpha_\omega(\mathbf{\Theta} \to \mathbf{\Theta}') = \min\left\{1, \frac{P(\mathbf{D}|\mathbf{\Theta}')}{P(\mathbf{D}|\mathbf{\Theta})}\exp\left(-\lambda_\omega\left(\omega_i' - \omega_i\right)\right)\frac{\omega_i'}{\omega_i}\right\}. \tag{21}$$

Here, $L(\mathbf{\Theta}|\mathbf{D})$ is the likelihood of $\mathbf{\Theta}$ given the data, $P(x)$ is the prior on x, and $q(x \to x')$ is the proposal distribution. The MCMC move to alter μ is identical.

A truncated normal, gamma, or uniform prior can also be chosen as a prior for ω_i in our program.

Recombination Probabilities r_i. Assuming independence between sites, and a distinct recombination rate at each site, we must vary r_i for $i \in \{2, 3, \ldots, m\}$, where m is the number of codon sites in the sequence. We do this by perturbing the recombination rate at a given site in exactly the same way as for the selection parameters (ω_i) through a shift on the log scale. The recombination rate is related to the probability of recombination through the relation

$$r_i = 1 - \exp(-\nu_i/k), \tag{22}$$

where ν_i is the recombination rate between sites $i-1$ and i, and k is the number of sequences. We allow moves to alter the recombination rate in the same way as the selection parameters, ω_i. A new value ν' is chosen such that $\nu' = \nu \exp(U)$, where $U \sim \text{Uniform}(-1, 1)$. We have an exponential prior for ν with rate parameter λ_ν. The acceptance probability is identical to that of the selection parameters, with ω_i and λ_ω replaced with ν_i and λ_ν respectively.

Window Moves. Here, we consider only escape parameters (moves associated to reversion parameters are completely analogous) and for notational convenience we let $\gamma_{h,i} = \gamma_i$, and allow i to now enumerate windows rather than sites.

1. **Changes of selection parameters**

   Moves are proposed in the same way as for selection parameters, ω_i, but across a window rather than at a specific site. The window is chosen uniformly at random from the current collection of windows. We use a log-normal distribution, $\log \mathcal{N} \sim (0, \sigma_\gamma^2)$ for the prior on HLA dependent scaling of selection parameters, and the resulting acceptance ratio is

$$\alpha_{w_1}(\mathbf{\Theta} \to \mathbf{\Theta}') = \min \left\{ \frac{P(\mathbf{D}|\mathbf{\Theta}')}{P(\mathbf{D}|\mathbf{\Theta})} \exp\left(\frac{\log^2 \gamma_i - \log^2 \gamma_i'}{2\sigma_\gamma^2} \right) \frac{\gamma_i'}{\gamma_i} \right\}. \tag{23}$$

2. **Extension of a window in the $3'$ or $5'$ direction**

   Choose a window to extend uniformly at random, with $5'$ or $3'$ direction chosen with equal probability. Choose the number of sites to extend $g \in [1, \infty)$ from a geometric distribution. Any proposal that results in a window being merged is rejected, as is the proposal to extend the $5'$-most or $3'$-most block in the $5'$ or $3'$ direction respectively. Otherwise, the acceptance probability is

$$\alpha_{w_2}(\mathbf{\Theta} \to \mathbf{\Theta}') = \min \left\{ 1, \frac{P(\mathbf{D}|\mathbf{\Theta}')}{P(\mathbf{D}|\mathbf{\Theta})} \right\}. \tag{24}$$

3. **Split a window**

   These final two moves are complementary reversible jump MCMC moves. A site s^* is chosen uniformly from the collection of available splitting sites, and with probability 1 splits an existing window, $[s_i, s_{i+1})$ say. New HLA dependent selection parameters within these new selection windows $[s_i, s^*)$ and $[s^*, s_{i+1})$ are defined as γ_{i_1}' and γ_{i_2}' where

$$\gamma_{i_1}'^{\,(s^*-s_i)} \gamma_{i_2}'^{\,(s_{i+1}-s^*)} = \gamma_i^{(s_{i+1}-s_i)}, \tag{25}$$

$$\frac{\gamma_{i_2}'}{\gamma_{i_1}'} = \frac{1-u_1}{u_1}; \quad u_1 \sim U[0,1]. \tag{26}$$

   It then follows that the acceptance ratio is

$$\alpha_{w_3}(\mathbf{\Theta} \to \mathbf{\Theta}') = \min \left\{ 1, \frac{P(\mathbf{D}|\mathbf{\Theta}')}{P(\mathbf{D}|\mathbf{\Theta})} \frac{p_w}{1-p_w} \frac{\gamma_i}{\gamma_{i_1}' \gamma_{i_2}' \sqrt{2\pi}\sigma_\gamma} \right.$$
$$\left. \times \exp\left(\frac{\log^2 \gamma_i - \log^2 \gamma_{i_1}' - \log^2 \gamma_{i_2}'}{2\sigma_\gamma^2} \right) \frac{d_{S+1}}{c_S} \frac{m-S-1}{S+1} \frac{(\gamma_{i_1}' + \gamma_{i_2}')^2}{\gamma_i} \right\}, \tag{27}$$

where

$$c_S := \min\left\{1, \frac{P(S+1)}{P(S)}\right\} \quad \text{and} \quad d_S := \min\left\{1, \frac{P(S-1)}{P(S)}\right\} \tag{28}$$

are the probability of proposing a split and merge move respectively when the current number of splitting sites is S.

4. **Merge a window**. Pick a splitting site s_{i+1}, $i \in \{0, 1, \ldots, S-1\}$ at random to remove, resulting in a merge of windows $[s_i, s_{i+1})$ and $[s_{i+1}, s_{i+2})$. Let

$$\gamma'^{(s_{i+2}-s_i)}_i = \gamma_i^{(s_{i+1}-s_i)} \gamma_{i+1}^{(s_{i+2}-s_{i+1})}, \tag{29}$$

and accept with probability

$$\alpha_{w_4}(\Theta' \to \Theta) = \min\left\{1, \frac{P(\mathbf{D}|\Theta')}{P(\mathbf{D}|\Theta)} \frac{(1-p_w)}{p_w} \frac{\gamma_i \gamma_{i+1} \sqrt{2\pi}\sigma_\gamma}{\gamma_i'} \right.$$
$$\left. \times \exp\left(\frac{\log^2 \gamma_i + \log^2 \gamma_{i+1} - \log^2 \gamma_i'}{2\sigma_\gamma^2}\right) \frac{c_{S-1}}{d_S} \frac{S}{m-S} \frac{\gamma_i'}{(\gamma_i + \gamma_{i+1})^2} \right\}. \tag{30}$$

A truncated Normal, Gamma, or uniform prior can also be chosen as a prior for the escape and reversion parameters in our software.

Simulated annealing during burn-in

As part of the burn in period during MCMC runs, we perform simulated annealing. This allows us to explore the parameter space as much as possible during these early stages. Doing so is very simple within an MCMC framework. At earlier steps in the MCMC the chain is 'hotter'. This simply means that the likelihood portion of the posterior carries less weight in the posterior and thus flattens the posterior density, allowing for greater exploration of the parameter space. We do this by raising the likelihood ratio to the power T where $T > 1$. Note that, as $T \to \infty$ we are effectively sampling from the prior. We then let T be a function of the MCMC step t, such that $T(t) \to 1$ as $t \to \infty$. The functional form that we choose is

$$T : \mathbb{N} \to \mathbb{R}, \qquad T(t) = 1 + (T_0 - 1)\alpha^t. \tag{31}$$

Note that $T(0) = T_0$. We set $\alpha = 0.999$, and $T_0 = 500$.

Simulation study 2: methods comparison

Six methods are applied to the sequence and HLA information simulated under 100 independent birth-death processes (described in the Methods subsection Simulation study 2: A sampled birth death process):

1. Fisher's exact test (as in Moore *et al.* [6]).
2. Phylogenetically corrected Fisher's exact test (as in Bhattacharya *et al.* [7]).
3. Approximate escape rate estimate (as in Fryer *et al.* [8]).
4. 'Phylogenetic dependency networks' approach - PhyloD (as in Carlson *et al.* [9]).
5. PhyloD OR (as in Carlson *et al.* [10]).
6. Our methodology as described in the Methods section of the main text.

In each case, we assume that the consensus 'wild type' strain is correctly defined and set any non-synonymous difference from consensus as escape. For methods 2, 4 and 5, we determine a maximum likelihood estimate of the underlying genealogy using RAxML version 8.2.11 [11]. Methods 1, 3 and 6 do not require an estimate of the genealogy. We now briefly summarise each of the compared methods.

Fisher's exact test A simple Fisher's exact test is applied by splitting the query sequences according to the following 2×2 contingency table, from which p-values are determined at each site and each HLA type, independently.

	Wild-type	Escape
HLA matched	a	b
HLA mismatched	c	d

Phylogenetically corrected Fisher's exact test We use the methods of Bhattacharya *et al.* to help correct for the underlying dependency structure inherent in the underlying genealogy. Briefly, maximum likelihood estimates are obtained for the the most MRCA of all leaves in the estimated maximum likelihood tree. p-values are then obtained using the following 2×2 contingency table for each site and each HLA type, independently.

	Wild-type → Wild-type	Wild-type → Escape
HLA matched	a	b
HLA mismatched	c	d

Approximate escape rate estimate Assuming the model of Fryer *et al.* [12] has reached stationarity in the exponential growth phase, Fryer *et al.* write down simple formulae to determine estimates of host population rates of escape and reversion.

$$\lambda_{\text{esc}} = \beta c(1 - f_{\text{HLA}}) \left(\frac{\Lambda^1 - \Lambda^0}{1 - \Lambda^1} \right); \quad \lambda_{\text{rev}} = \beta c f_{\text{HLA}} \left(\frac{\Lambda^1 - \Lambda^0}{\Lambda^0} \right), \tag{32}$$

where λ_{esc} and λ_{esc}, β, c and f_{HLA} are estimates of the population escape and reversion rates, transmission probability, contact rate, and proportion of individuals in the population with the HLA type under investigation. Λ^0 and Λ^1 are the proportion of HLA mismatched hosts with an escape mutant at the investigated site, and proportion of HLA matched hosts with an escape mutant at the investigated site. We note that neither β nor c are required to preserve the ordering of escape and reversion rates, and as such do not affect the associated ROC curves. The ordering is also preserved at the stationary phase [8]. In cases where the resultant escape or reversion rate estimate is negative, we set the escape rate estimate at zero under the assumption that the model does not fit the simulated data.

PhyloD We implement the methods of Carlson *et al.* as described in [9]. As described above, we determine an estimate of the maximum likelihood tree, $G_{\max}$ using RAxML [11] before using likelihood ratio tests to compare models conditional on the maximum likelihood tree. The initial null model at each site is a simple two state model described by two parameters - the mutation rate; λ, and the stationary probability of observing the wild-type amino acid; π. The associated instantaneous rate matrix is

$$Q = \begin{array}{c} \\ \text{wild-type} \\ \text{escape} \end{array} \begin{array}{c} \begin{array}{cc} \text{wild-type} & \text{escape} \end{array} \\ \begin{pmatrix} -\lambda\pi & \lambda\pi \\ \lambda(1-\pi) & -\lambda(1-\pi) \end{pmatrix} \end{array}. \tag{33}$$

Felsenstein's peeling algorithm [13] is then used to evaluate the likelihood $L(\lambda, \pi | D, G_{\max})$ and maximised, where D is the combination of simulated query sequences and associated host HLA data. So called 'leaf-distributions' are then potentially added using forward selection. This involves the inclusion of further hidden states - estimates of the transmitted sequence for each subsequently sampled member of the query sequences set. This transmitted sequence is then potentially modified conditional on the hosts' HLA. In the case of escape, the probability of an instantaneous switch from wild-type to escape is parameterised by a probability; p. Thus, given Q above for the remainder of

the tree, and probabilities of switching between states between the hidden 'transmitted sequence' and the query sequences at the leaves, we can evaluate $L(\lambda, \pi, p|D, G_{\max})$ using tree peeling. Maximising this likelihood and comparing to the current null model, parameters are added if the p-value for the associated likelihood ratio test is lower than 0.05. Whereas in Carlson *et al.* maximum likelihood parameterisations are obtained by either coordinate descent [14] or expectation-maximisation [9], we take a pragmatic approach, as often certain optimisation schemes fail under different scenarios. To this end, we simultaneously run six different optimisation schemes built into the optimx R package [15]: Nelder-Mead [16], L-BFGS-B [17], ucminf [18], nmkb [19], newuoa [20], bobyqa [21]. We stipulate that at least two schemes must converge, and choose the likelihood parameterisation of the scheme with the highest likelihood. If less than two schemes converge, new starting parameters are chosen at random and estimates are re-evaluated.

PhyloD OR A slight modification of PhyloD, PhyloD OR also involves a simple alteration of the standard peeling algorithm, but instead of a 'leaf-distribution' being added at the leaves, a logistic regression is applied between the hidden transmitted states and the observed sequence data at the leaves - see Carlson *et al.* for some details [10]. Likelihoods can then be evaluated based on the probabilities associated to this logistic regression fit using a peeling algorithm [13]. E.g. Rearranging

$$log\left(\frac{P}{1-P}\right) = \sum_i a_i X_i + cT \tag{34}$$

$$\Rightarrow P = \frac{\exp\left(\sum_i a_i X_i + cT\right)}{1+\exp\left(\sum_i a_i X_i + cT\right)}, \tag{35}$$

where P is the probability of observing an escape mutation, X_i are binary variables taking the value 0 or 1 (for presence/absence of HLA i) depending on the HLA status of the host associated to a given leaf sequence. T is a further binary variable, which takes the value -1 if the state at the hidden transmitted state is wild-type, and 1 if it is escape. Given that these transmitted states are unknown, they are summed over using tree peeling. a_is and c are fitted using maximum likelihood optimisation schemes as for PhyloD. Following Carlson *et al.* we again use forward selection and likelihood ratio tests and add parameters to the model if $p < 0.05$.

Using each of the five methods described above, plus our new approach as described in detail in the methods section of the main text, we obtain p-values or parameter estimates which provide a metric for the strength of selection for escape at each site, conditional on each of the HLA types. We use the p-value, p-value, escape rate estimate, probability estimate, strength of selection; a_i, and median HLA associated selection parameters; γ_h for each of the six methods respectively to determine ROC curves for each of the 100 simulated sequence and host HLA data sets, displayed in Figure 2 of the main text. We also examined the effect of increasing the query sequence set with associated host HLA information to 3000. The resultant ROC curves for each of the five methods are shown in Supplementary Figure 7.

To examine the impact of a reduced reference data set $\mathbf{D}_\mathrm{B}$ upon parameter estimates, we subset to 100%, 10% and 1% of the reference sets in Simulation Study 2. We then performed inference and determined the impact of a smaller $\mathbf{D}_\mathrm{B}$ on the accuracy of our results. ROC curves for our method with different reference data sets sizes are shown in Supplementary Figure 8.

Query and reference data set preparation

Drug associated selection analysis

All sequences and their associated host drug regime data were downloaded from the Stanford drug resistance database [22]. We used the alignments provided, though sites at which there was ≥5% ambiguity were removed, followed by sequences for which there was ≥5% nucleotide ambiguity across

the region of interest. In *reverse transcriptase* a ∼550 nucleotide region is sequenced much more frequently than the remainder of the gene and we wish to retain power to estimate parameters. One sequence per patient was included in the combined alignment of reference and query data sets by removing repeat entries of any given patient identifier uniformly at random (assuming unique patient identifiers ⇒ unique patients).

The data set was then split in two, sequences for which the host was not receiving any drugs $\mathbf{D}_{\mathrm{B}}$, and sequences for which the host was receiving drugs $\mathbf{D}$. 1,000 sequences were then randomly chosen from $\mathbf{D}_{\mathrm{B}}$ and placed in $\mathbf{D}$. As described in the text, this step was included to guarantee variation within the query data set in drug associated selection. $\mathbf{D}_{\mathrm{B}}$ defines our reference data set. We perform some further data cleaning steps to create the query data set from $\mathbf{D}$.

Sequences were removed from $\mathbf{D}$ if RTI, NNRTI, NRTI or PI was used as a descriptor of one of the drugs in a patients drug regime, as this was not specific enough for our purposes. Finally, any sequences which were associated to a drug present in $\mathbf{D}$ fewer than 10 times were removed. Drug information associated to the query sequences was reformatted into a .csv file with binary entries so that it could be read by our program.

The resulting collections of reference and query data sets consisted of 49,721 reference sequences and 6,130 query sequences with associated drug regime data for the *protease* data set, and 54,663 and 13,885 query sequences with associated drug regime data for the *reverse transcriptase* data set.

HLA associated selection analysis

To investigate HLA-associated selection was more cumbersome, we wished to incorporate as much data as was publicly available into our analyses. To achieve this, we focused on the most highly sequenced portions of the viral genome: *protease* and *reverse transcriptase.* To create the reference data sets we concentrated on three major sequence resources:

1. Los Alamos HIV sequence database.
2. Stanford Drug resistance database.
3. UCLA HIV positive selection mutation database.

Before filtering, the combination of these three databases represented a total of $119,878 + 81,533 + 45,161 = 246,572$ and $148,866 + 88,780 + 45,161 = 282,807$ sequences in *protease* and *reverse transcriptase* respectively. For the Los Alamos HIV sequence database portion of the *reverse transcriptase* reference data set, we allow sequence chunks of length >500 nucleotides.

To create the query data sets, we combined viral sequence data with associated host HLA information from the following sources (see also Table 1 in the main text):

- Swiss Spanish intermittent treatment trial (SSITT) data set [23, 24]: Swiss portion of this data set.
- Durban data set [25, 26].
- Mma Bana (mother and child) study [27]: adult portion of this study.
- Bloemfontein data set [28, 29].
- The short pulse antiretroviral therapy at seroconversion (SPARTAC) data set [30]: UK portion of this data set.
- All remaining sequences with associated host HLA data available in the Los Alamos HIV sequence database [31] which were not present in the above studies.

In order to arrive at the final collection of reference and query sequences combined with HLA information we applied a number of filtering steps.

Creating the reference sequence data set.

Filtering steps

1. Remove overlap between the Stanford drug resistance database and Los Alamos HIV sequence database: for any patient identifiers present in both databases, we remove all sequences corresponding to that patient from the Los Alamos database sequences.
2. Remove sequences with associated host HLA information from both the Stanford drug resistance database and Los Alamos HIV sequence database sequences: this was achieved by comparing patient identifiers. The appropriate sequences were removed from the Stanford drug resistance database and Los Alamos HIV sequence database sequences.
3. Remove repeat patient entries: again, this was achieved using patient identifiers. For any repeated patient identifier, one sequence was kept (chosen uniformly at random from among the sequences with the same patient identifier).

Sequence alignment

We next aligned the collection of sequences; a non-trivial task given the vast amount of sequence data. The alignment of *protease* in the Stanford drug resistance database is well maintained, so we assumed that this portion of the alignment is accurate. Note that this is why we remove the Los Alamos database copy where there is overlap between the Stanford drug resistance database and Los Alamos sequence databases. In the case of the sequences taken from the Los Alamos HIV sequence database, we find that attempting to generate an alignment when downloading ∼70,000 sequences yields nonsensensical results. However, there is a lack of indels in the *protease* region. We therefore simply considered sequences of exactly 297 nucleotides for the alignment (after removal of gaps from the alignment proposed using the Los Alamos database default alignment software). Performing this step led to a loss of <500 sequences out of a total of ∼70,000. We then checked the resulting distribution of nucleotides across the region. Finally, the UCLA positive selection database was assumed to be aligned correctly.

Such a simple alignment procedure was not available to us for the *reverse transcriptase* sequences due to the increased prevalence of indels within *reverse transcriptase*, coupled with the longer sequence length. We reduced the size of the collection of sequences to be aligned by assuming sequences taken from the Stanford drug resistance database were correctly aligned. Aligning all the remaining sequences at once using MUSCLE [32] was prohibitive computationally, as was using the align option in MUSCLE (which can be used to combine alignments: this procedure also tended to produce artefacts in the overall alignment). We therefore considered the approach of aligning subsets of 5000 sequences and comparing these sub-alignments. We found that although more common than within *protease*, indels are rare. Removing them resulted in a collection of sub-alignments that aligned with both each other and the consensus sequences for both the Stanford drug resistance database and UCLA positive selection database alignments.

Quality control

We removed ambiguous codons and sequences from both the *protease* and *reverse transcriptase* alignments by first deleting codons with $> 5\%$ ambiguity and then sequences with $> 5\%$ sequence ambiguity as in our data set prepartion for the drug associated selection analysis. Removal of sequence ambiguity resulted in a total of $162,901$ and $184,817$ reference sequences for *protease* and *reverse transcriptase* respectively.

Creating the query data set

Filtering steps

As in the reference sequence filtering step, we remove repeat patient entries using patient identifiers: For any repeated patient identifier, one sequence was kept (chosen uniformly at random from among the sequences with the same patient identifier).

Sequence alignment

We aligned the query sequences using MUSCLE [32]. We found that no gaps were introduced (aside from at the beginning and end of the sequences). Both the *protease* and *reverse transcriptase* query sequences align with their respective reference sequence data sets.

Quality control

We checked ambiguity of codons and sequences within the query data sets using the same criteria as in our reference data set preparation, removing codon positions and sequences as required.

Throughout, HLA typing was restricted to two digits and HLA-A, B, and C alleles were analysed jointly. We truncated to two-digits in the interests of power: although the most common 4 digit HLAs could be considered separately, the majority are rare at 4 digit resolution in our data set (60.5% of 4-digit HLAs have a count of < 10, compared to 30.2% at 2-digit resolution). Moreover, 27.3% of the individuals were only typed at 2-digit resolution. However, this should be feasible in the future as sample sizes of viral sequence data with associated host HLA types increase.

In both the query and reference data sets after filtering we obtain a relatively constant sequence ambiguity across the newly defined regions of *protease* and *reverse transcriptase*, with slight increases at the start and end of the region as we would expect. Consensus nucleotide proportion across both newly defined regions averaged across all sites is 0.941 and 0.943 for *protease* and *reverse transcriptase* respectively, with any large drop below this coinciding with a subtype B/C consensus sequence difference at the nucleotide level.

The resulting cleaned data sets consist of:

- A reference data set $\mathbf{D}_\mathrm{B}$ consisting of sequences taken from Los Alamos sequence database, the Stanford Drug resistance database and the UCLA positive selection database for which no host HLA information was available.
- A query data set which consists of:
  1. Viral sequence data $\mathbf{D}$.
  2. Host 2 digit HLA genotype data $\mathcal{H}$ for the three HLA class I alleles associated to the viral sequences in $\mathbf{D}$.

Dendrograms of selection profiles and comparing topologies

We determine whether HLA alleles with similar sequences, or drug with similar modes of action lead to selection responses that are similar. To obtain dendrograms of drug selection profiles and HLA selection profiles we first assign estimated per-site selection coefficients to one of four broad classes, based on the median of the selection profile at that site:

Selection class	0	1	2	3
Selection coefficient	$\gamma \leq 1$	$1 < \gamma \leq 1.5$	$1.5 < \gamma \leq 2$	$\gamma > 2$

We avoid considering lower quantiles due to potential biases which would be induced by drug/HLA specific sample sizes. We then perform hierarchical clustering on assigned selection classes using `hclust` (in the R stats library), using the complete linkage method. The resultant dendrograms for drug associated selection are shown in Supplementary Figure 13.

We compare the topology of dendrograms obtained from HLA selection profiles to dendrograms obtained from human HLA protein sequence information from the IMGT database [33]. To obtain a dendrogram based on host HLA sequence information for comparison, we first remove identical HLA protein sequences in the available sequenced region and restrict analysis to the first four members of each 2-digit HLA type for which we have estimated an HLA selection profile for HIV-1. We use RaxML [11] to obtain maximum likelihood trees for each of the three class I molecules. As we expect, members of the same 2-digit HLA type cluster together.

To compare dendrogram topologies, we determine the number of 2-digit HLA types which share closest neighbours (closest leaf within the dendrogram) across the 2 dendrogram topologies. As a permutation test, we shuffle tip labellings to obtain a distribution on this metric. As HLA alleles with the same first two digits lie at slightly different positions in the host HLA protein dendrogram, we randomly restrict to one representative of each 2-digit HLA 100 times, and perform 10,000 label shuffles for each of these restrictions to obtain the distribution for our nearest neighbour metric. From this distribution, we calculate p-values and odds ratios.

Supplementary Notes

Derivation of expected divergence time to closest relative in a coalescent tree with k leaves

Consider adding a new lineage to an existing tree with k lineages. We are interested in the the distribution of the time, t, at which it first coalesces with an ancestral lineage within the existing genealogy. To derive the mean, let T_k be the total tree length of a coalescent tree with k lineages. Then,

$$\mathbb{E}[T_k] = \sum_{i=2}^{k} i\mathbb{E}\left[Exp\left(\binom{i}{2}\right)\right] \tag{36}$$

$$= \sum_{i=2}^{k} \frac{i}{\frac{i(i-1)}{2}} = 2\sum_{i=2}^{k} \frac{1}{i-1}. \tag{37}$$

Thus, since $t = T_{k+1} - T_k$,

$$\mathbb{E}[t] = \mathbb{E}[T_{k+1}] - \mathbb{E}[T_k] \tag{38}$$

$$= \frac{2}{k}, \tag{39}$$

To approximate the distribution of the time to coalescence between a lineage and the rest of the tree, we use an exponential distribution with parameter $\frac{k}{2}$.

Comparison of results at well-studied epitopes

We compare inference within our study (summarised in Figure 5b) to previous results at a series of well-studied epitopes. Putative CTL escape sites within HLA-B associated A-list epitopes are highlighted in yellow. Notice that many A-list epitopes are not variable.

*B*07 epitope* `SPAIFQSSM` *(sites 156-164 in reverse transcriptase).*
We detect strong B*07-dependent selection at the known escape site (position 162) [34, 35]. We also find two more top-tier sites and several second-tier sites within the epitope. On further investigation of the literature we find that many variants exist for this epitope, which result in non-susceptible forms in some individuals and susceptible forms in others [36].

*B*18 epitope* `NETPGIRYQY` *(sites 137-146 in reverse transcriptase).*
We detect strong B*18-dependent selection at the first anchor residue of `NETPGIRYQY` (site 138) [35, 37]. We do not detect selection at the other escape site. Further investigation of the literature reveals that the 'diminished response' induced by a variant at this site is actually recognised to much the same levels as the wild type epitope, and precedes outgrowth of the single variant at the first anchor residue in the single patient studied [38].

*B*35 epitope* `HPDIVIYQY` *(or* `NPDIVIYQY`*, sites 175-183 in reverse transcriptase).*
We do not detect B*35-dependent selection in this epitope when considering all of our query sequences. However, we identify second-tier sites within `HPDIVIYQY` when considering only subtype C sequences in our query data set and setting $\mathscr{C}$ as the subtype B consensus sequence.

*B*40 epitope* `IEELRQHLL` *(sites 202-210 in reverse transcriptase).*
We do not detect B*40-dependent selection in `IEELRQHLL`. B*40 is a relatively rare HLA type in our query data sets and particularly rare in hosts with subtype C viral sequences (<10 sequence/host HLA pairs have B*40).

*B*44 epitope* `EEMNLPGRW` *(sites 34-42 in protease).*
Moving on to protease in Supplementary Figure 15, we detect a strong signal of selection within the

B*44 epitope `EEMNLPGRW`. This signal is particularly strong at the first anchor residue (site 35) where escape is known to occur [39, 40].

We now consider well-studied HLA-A epitopes, again starting with reverse transcriptase. See Supplementary Figures 17 (note there are no HLA-C epitopes in our well-studied list):

*A*02 epitope* `YTAFTIPSV` *(sites 127-135 in reverse transcriptase).*
We do not detect A*02-associated selection in `YTAFTIPSV`. The subtype B and C consensus residue at the escape site (position 135) is the defined escape allele [41], explaining why we do not detect a signal. However, we notice that a signal at the escape site is picked up when considering subtype B viruses (see the fifth and sixth A*02 strips of Supplementary Figure 17). This suggests that other mutations at the same residue can also result in a diminished CTL response.

*A*03 epitope* `AIFQSSMTK` *(sites 158-166 in reverse transcriptase).*
Escape is known to occur at the second anchor residue (site 166) [42, 43]. This site is in our collection of second tier candidates, see Supplementary Figure 17, with much of the signal coming from the subtype C viruses. However, we do not detect any A*03-associated selection elsewhere in the epitope.

*A*11 epitope* `AIFQSSMTK` *(sites 158-166 in reverse transcriptase).*
We do not detect A*11 dependent selection in this epitope. A*11 is a relatively rare HLA allele in our query data set.

*A*02 epitope* `LVGPTPVNI` *(sites 76-84 in protease).*
Finally, looking at Supplementary Figure 14 we see that A*02-dependent selection is detected in `LVGPTPVNI`, but not at the reported escape site. We detect strong selection at the first anchor residue, and slightly upstream of the epitope. Interestingly, some literature suggests that the wild type sequence is not recognised, and a drug induced mutation at the escape site (position 82) results in epitope recognition [44]. These conflicting signals may explain why we do not detect A*02-associated selection at the known escape site.

These findings demonstrate the ability of our method to accurately identify many sites known to be under HLA associated selection, and highlight the nuanced effects of many putative escape variants.

Supplementary Figures

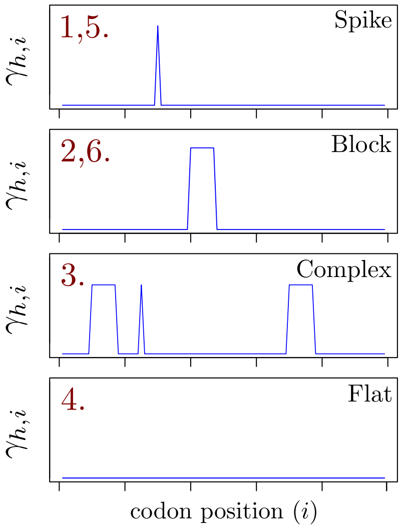

HLA	Profile	n
1	Spike	100
2	Block	100
3	Complex	100
4	Flat	100
5	Spike	30
6	Block	30

Figure 1: Simulation study 1: summary of the selection profiles used. Each subplot on the left-hand side indicates the HLA allele associated selection intensity away from the consensus codon, $\boldsymbol{\gamma}$, with the number denoting the name of that HLA. The table on the right indicates the HLA allele count in the simulated data for the HLA profiles shown on the left. Where HLA selection intensity exceeded 1, reversion ζ_i was set at $\frac{2}{3}$ this intensity, and 1 otherwise. Non-HLA-associated selection, ω_i, was sampled from $\log \mathcal{N}(0, 4)$. μ was set at 40, and κ was set at 7.60. The recombination probability between sites, r_i, was set at a constant across the region, which we varied between collections of runs ($\mathbf{r} \in \{\mathbf{0}, \mathbf{0.01}, \mathbf{0.05}\}$).

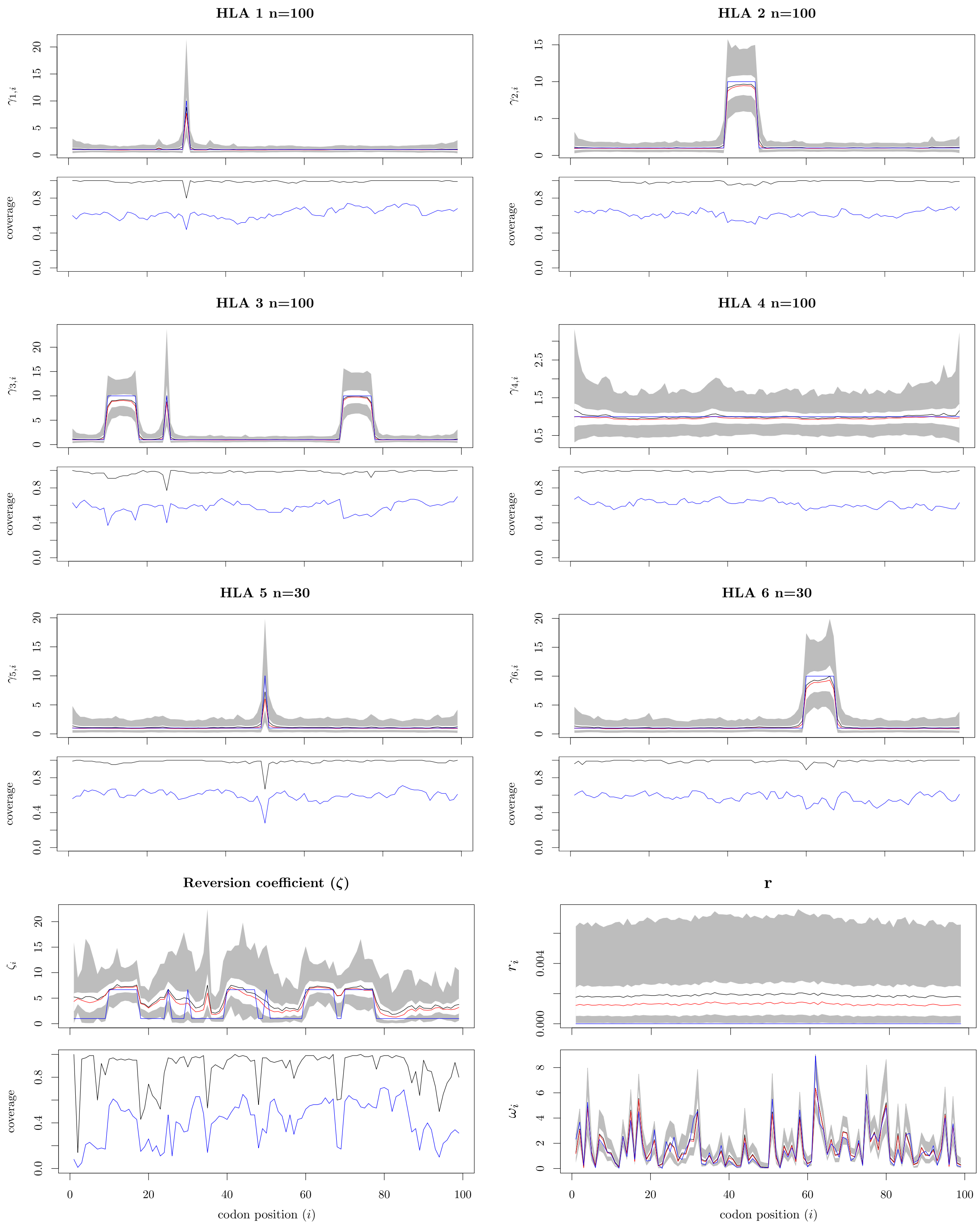

Figure 2: Simulation study 1: parameter estimates, no recombination ($\mathbf{r} = \mathbf{0}$). Odd rows show the average estimates and credible intervals. Averages are taken over 100 independent MCMC runs on independent simulated data with the same underlying parameters. The true underlying value is shown in blue, the mean and median estimates are displayed in black and white respectively. The 50% credible interval is enclosed by the white band, which is in turn enclosed by the grey 95% credible interval. Even rows (except for the final panel) show coverage plots for the two credible intervals: the black line tracks the proportion of the time the truth lies within the 95% credible interval for the 100 independent simulations and MCMC runs. The blue line tracks the proportion of runs in which the truth lies within the 50% credible interval. The final panel shows an example of inference of $\boldsymbol{\omega}$ across the region in one of the 100 simulations. Averages, truth, and credible intervals are coloured as in the odd rows. Source data are provided as a Source Data file.

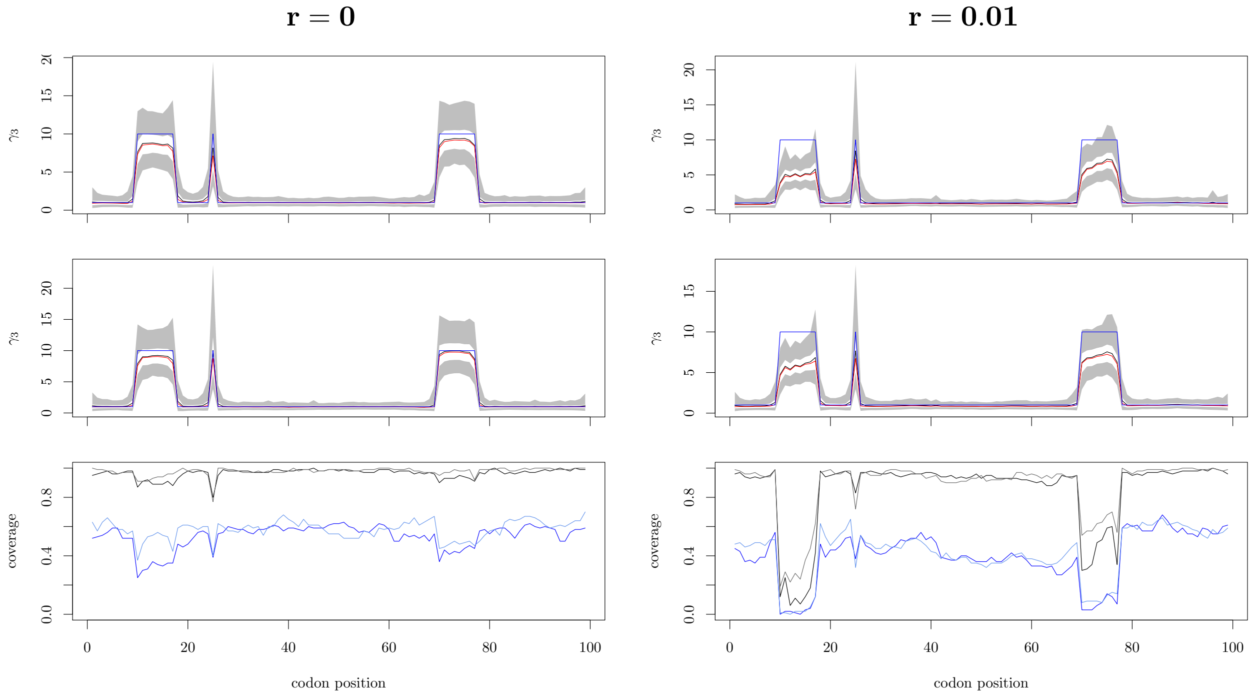

Figure 3: Simulation Study 1: comparison of estimates obtained using the closest 10 sequences by Hamming distance to the closest 100 sequences for $\mathbf{r} = \mathbf{0}$ and $\mathbf{r} = \mathbf{0.01}$. Averages are taken over 100 independent MCMC runs on independent simulated data with the same underlying parameters. The first two rows show results obtained using the closest 10 and 100 sequences by Hamming distance respectively. Columns show the results for $\mathbf{r} = \mathbf{0}$ and $\mathbf{r} = \mathbf{0.01}$ across the region respectively. The mean estimate is plotted in black, the median is plotted in red, and the true underlying parameter value is plotted in blue. The white band denotes the 50% credible interval within the 95% credible interval denoted by the grey band. The third panel displays the coverage. The black and grey lines display the proportion of the simulations for which the true underlying parameter lies within the 95% credible interval for each run when considering the closest 10 and 100 sequences by Hamming distance respectively. The dark and light blue lines display the proportion of the simulations for which the true underlying parameter lies within the 50% credible interval for each run when considering the closest 10 and 100 sequences by Hamming distance respectively. Source data are provided as a Source Data file.

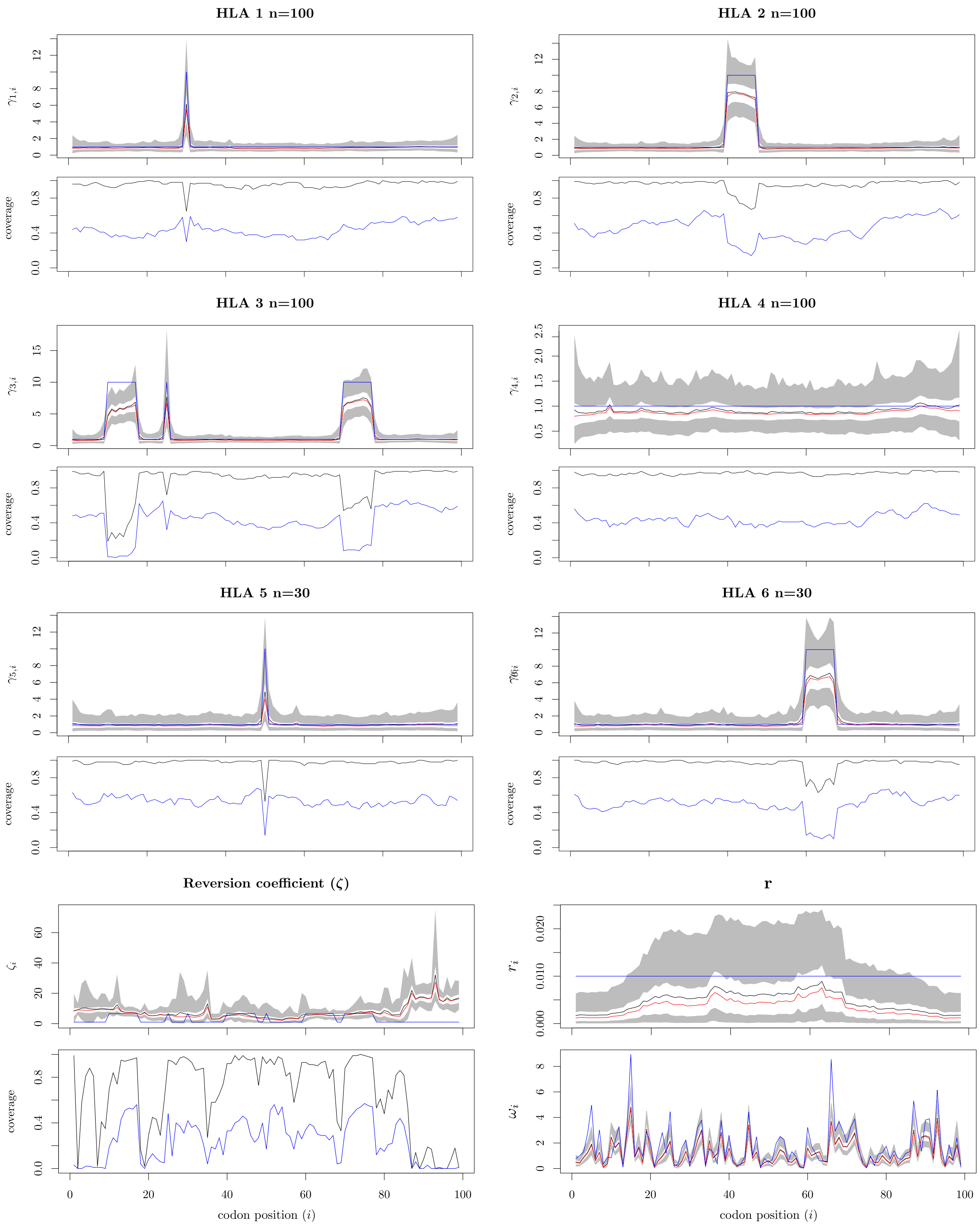

Figure 4: Simulation Study 1: parameter estimates, $\mathbf{r} = \mathbf{0.01}$. Odd rows show the average estimates and credible intervals. Averages are taken over 100 independent MCMC runs on independent simulated data with the same underlying parameters. The true underlying value is shown in blue, the mean and median estimates are displayed in black and white respectively. The 50% credible interval is enclosed by the white band, which is in turn enclosed by the grey 95% credible interval. Even rows (except for the final panel) show coverage plots for the two credible intervals: the black line tracks the proportion of the time the truth lies within the 95% credible interval for the 100 independent simulations and MCMC runs. The blue line tracks the proportion of runs in which the truth lies within the 50% credible interval. The final panel shows an example of inference of $\boldsymbol{\omega}$ across the region in one of the 100 simulations. Averages, truth, and credible intervals are coloured as in the odd rows. Source data are provided as a Source Data file.

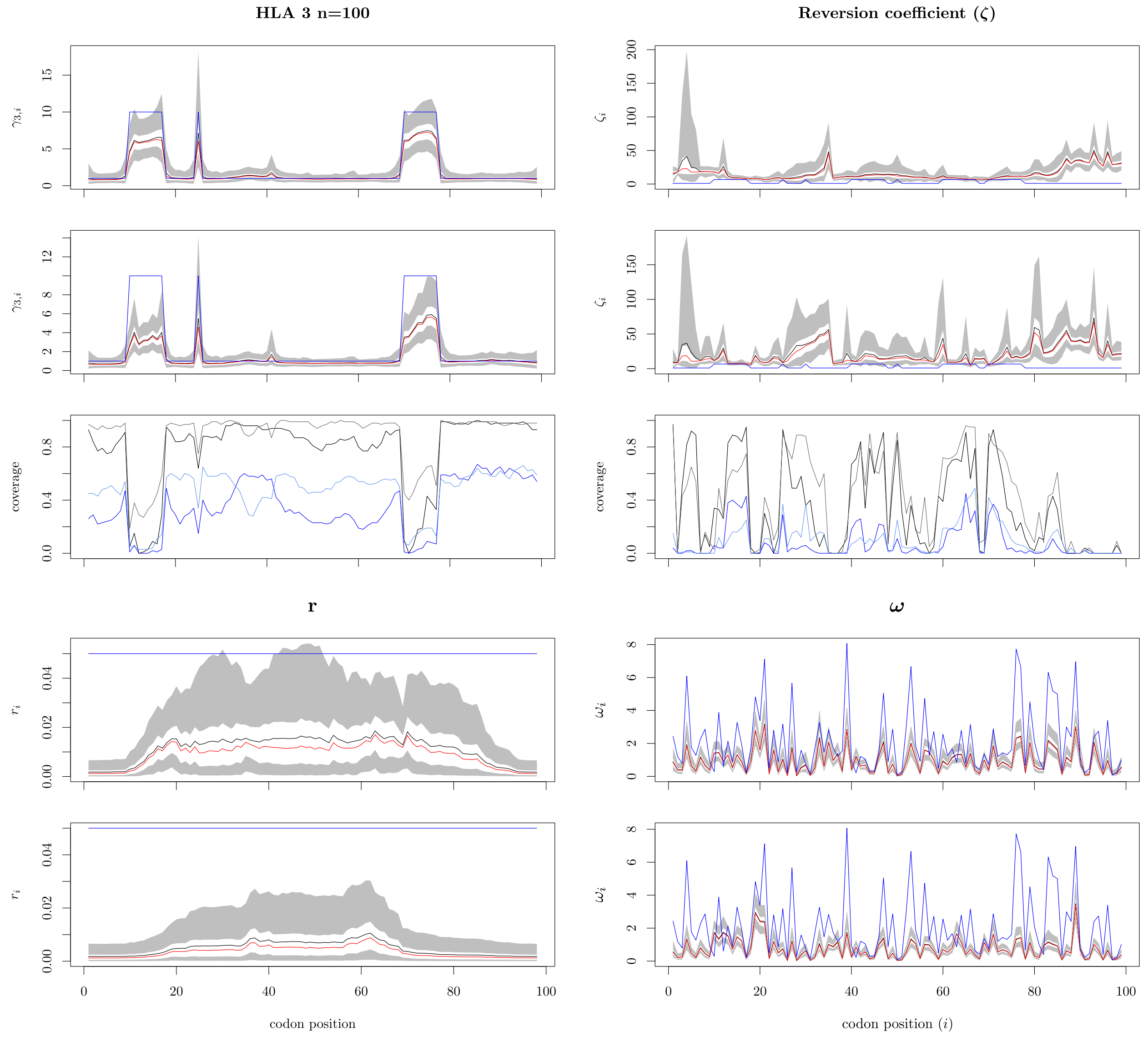

Figure 5: Simulation Study 1: comparison of estimates obtained using the closest 100 sequences by Hamming distance to the sequences actually copied from in the simulation, $\mathbf{r} = \mathbf{0.05}$. Averages are taken over 100 independent MCMC runs on independent simulated data with the same underlying parameters. Plotting for the first three rows is as in Figure 3. The first row shows inference results for HLA 3 associated selection and reversion using sequences copied from in the restriction of $\mathbf{D}_{\mathrm{B}}$, the second row shows the corresponding inference results when restricting $\mathbf{D}_{\mathrm{B}}$ is restricted using the closest 100 sequences by Hamming distance. The third row displays the coverage using the different restrictions. The final two rows display recombination estimates, and an example of inference of $\boldsymbol{\omega}$ across the region for the restriction using the sequences copied from and the closest 100 by Hamming distance respectively. Source data are provided as a Source Data file.

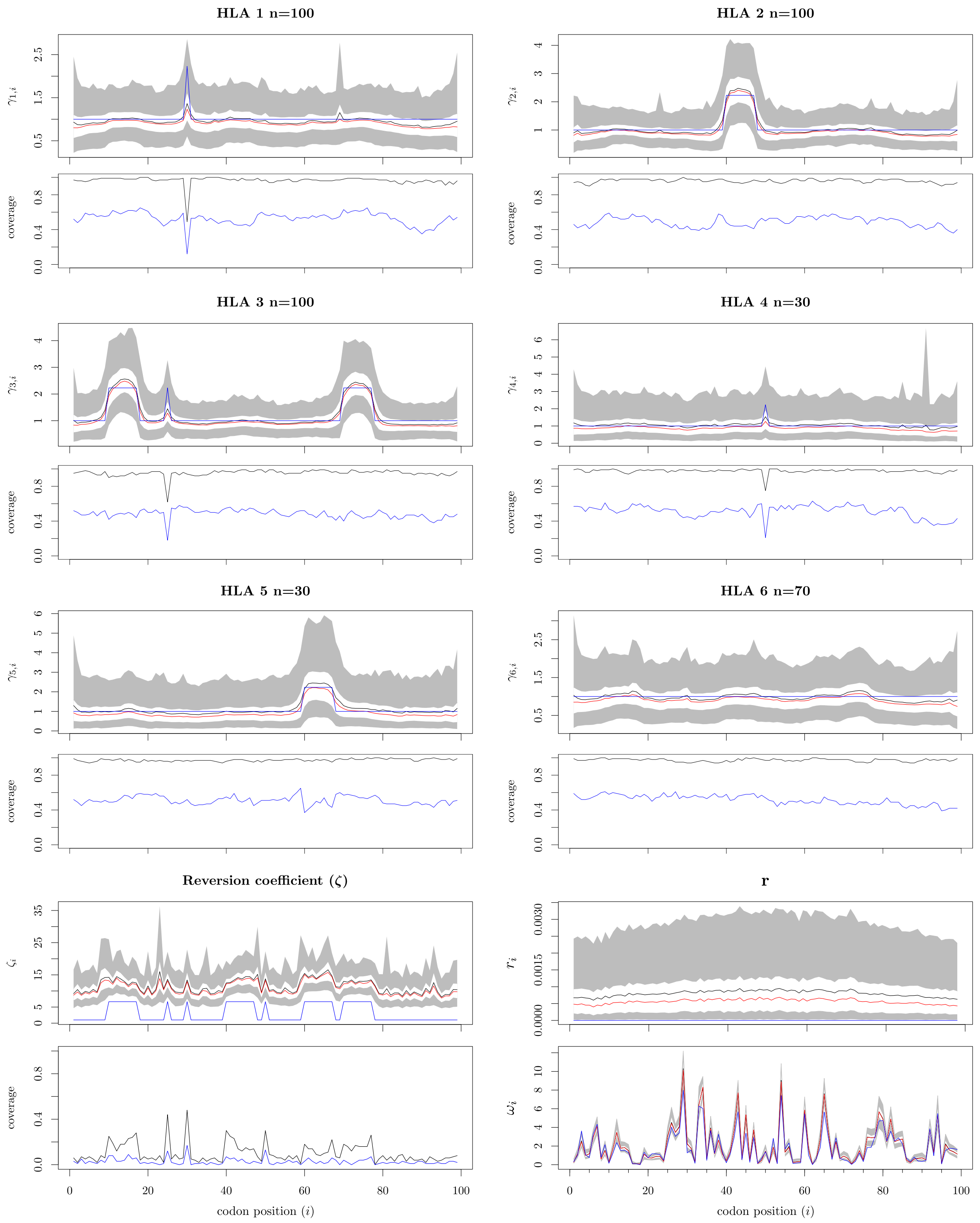

Figure 6: Simulation Study 2: parameter estimates. 100 independent birth-death processes were simulated. followed by 10% sampling and subsequent parameter inference. Estimates of HLA-associated scaling and posterior coverage are shown. Odd rows show the average estimates and credible intervals. Averages are taken over 100 independent MCMC runs on independent simulated data with the same underlying parameters. The true underlying value is shown in blue, the mean and median estimates are displayed in black and white respectively. The 50% credible interval is enclosed by the white band, which is in turn enclosed by the grey 95% credible interval. Even rows (except for the final panel) show coverage plots for the two credible intervals: the black line tracks the proportion of the time the truth lies within the 95% credible interval for the 100 independent simulations and MCMC runs. The blue line tracks the proportion of runs in which the truth lies within the 50% credible interval. The final panel shows an example of inference of $\boldsymbol{\omega}$ across the region in one of the 100 simulations. Averages, truth, and credible intervals are coloured as in the odd rows. Source data are provided as a Source Data file.

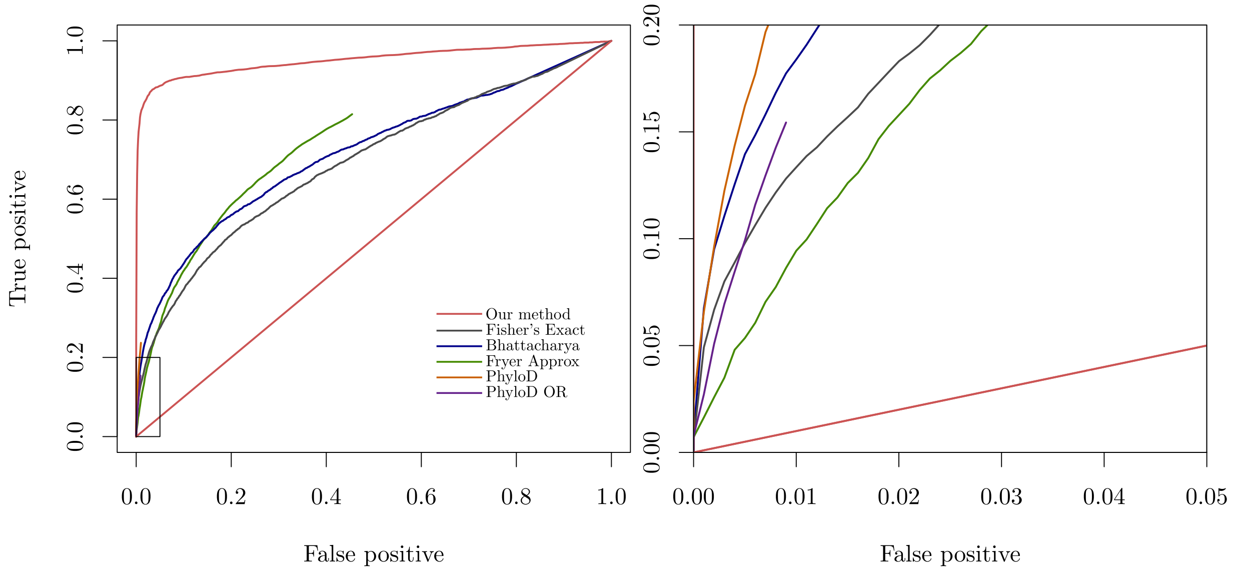

Figure 7: Simulation Study 2: sample size of 3000, results summary. Six methods used to identify HLA associated selection on viral sequence are applied to data simulated under the birth-death process used in simulation study 2. The second panel zooms in to the rectangle shown in the first panel. Coloured lines represent averages across 100 independent simulation runs. ROC curves for Fryer Approx, PhyloD and PhyloD OR do not extend to (1,1). For Fryer Approx this is because we stop our threshold for estimated rates at 0. For PhyloD and PhyloD OR, it is because many sites will not be included in leaf distribution or logistic regression respectively. Source data are provided as a Source Data file.

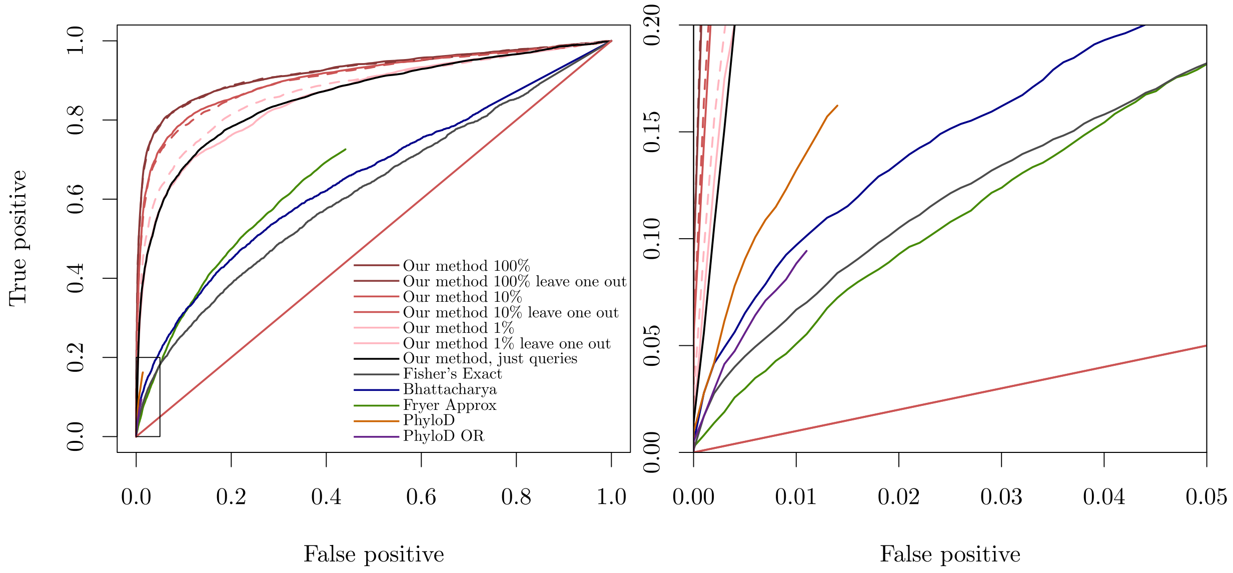

Figure 8: Simulation Study 2: impact of reduced reference sequence set, results summary. The second panel zooms in to the rectangle shown in the first panel. Coloured lines represent averages across 100 independent simulation runs. ROC curves for Fryer Approx, PhyloD and PhyloD OR do not extend to (1,1). For Fryer Approx this is because we stop our threshold for estimated rates at 0. For PhyloD and PhyloD OR, it is because many sites will not be included in leaf distribution or logistic regression respectively. Pink to red lines and dashed lines show the ROC curves for our approach, using different $\mathbf{D}_{\mathrm{B}}$. From light to dark, we sampled 100%, 10% and 1% of $\mathbf{D}_{\mathrm{B}}$ from each independent run in Simulation Study 2 and estimated HLA-associated selection. Dashed lines of the same colour also include $\mathbf{D}$ in $\mathbf{D}_{\mathrm{B}}$ using a leave one out approach. The black ROC curve shows the results of considering only $\mathbf{D}$ using a leave one out approach. Source data are provided as a Source Data file.

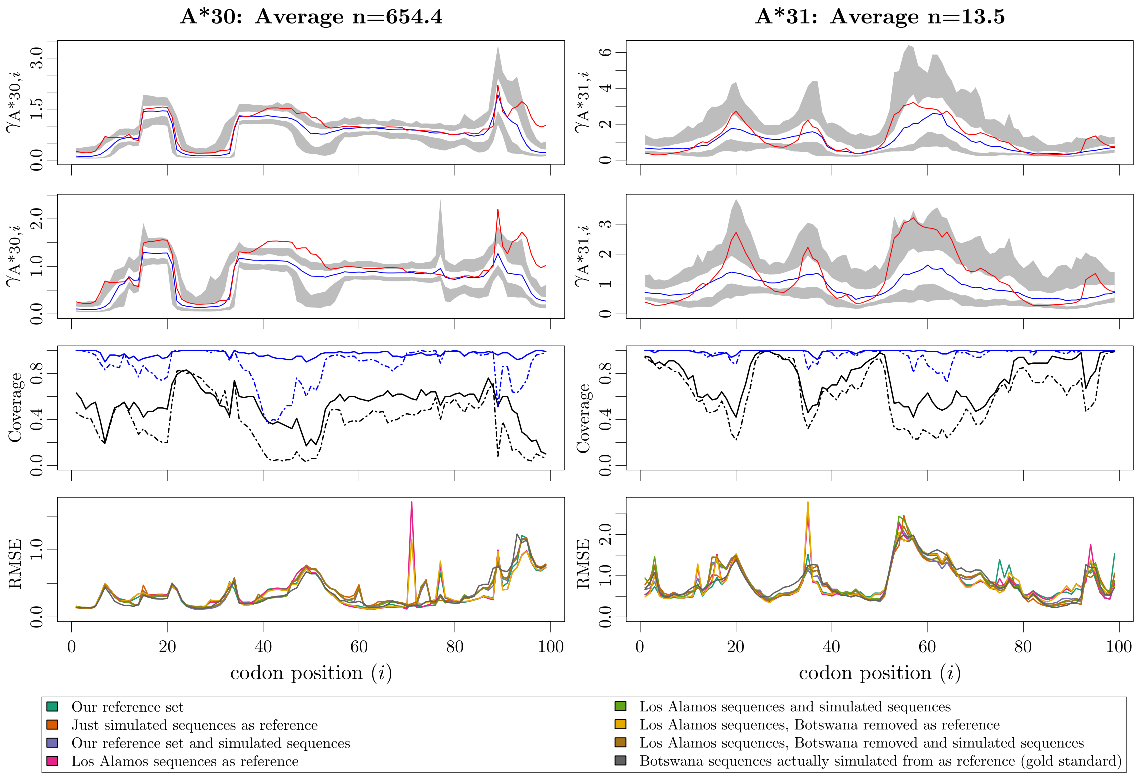

Figure 9: Simulation Study 3: example results for HLA-A alleles. We show results for one rare and one common allele for each of HLA-A. The first panel for each shows the gold standard - if we knew the sequences copied from to create $\mathbf{D}$ and included them in $\mathbf{D}_{\mathrm{B}}$. The second shows the results if we let $\mathbf{D}_{\mathrm{B}}$ be all sequences without associated host HLA information. The red line displays the true underlying HLA associated selection of the parametric bootstrap. The blue line displays the median estimate. Grey and white bands display the 5% – 95% and 25% – 75% credible intervals. In the third row, we display the coverage for the 95% and 50% credible intervals in blue and black respectively. The solid lines are using the gold standard reference set, and the dotted lines are using reference data from all public databases. In each of the fourth rows we display the average RMSE at each site for different $\mathbf{D}_{\mathrm{B}}$ as defined in the key. Source data are provided as a Source Data file.

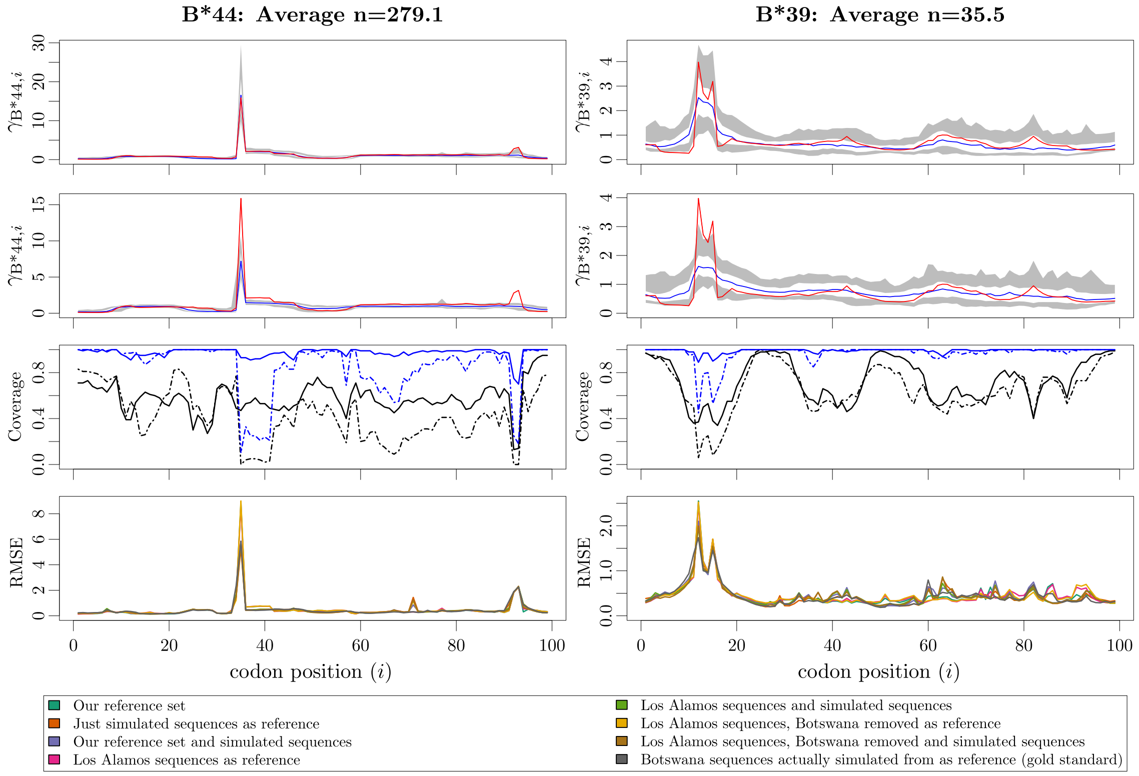

Figure 10: Simulation Study 3: example results for HLA-B alleles. We show results for one rare and one common allele for each of HLA-B. The first panel for each shows the gold standard - if we knew the sequences copied from to create $\mathbf{D}$ and included them in $\mathbf{D}_{\mathrm{B}}$. The second shows the results if we let $\mathbf{D}_{\mathrm{B}}$ be all sequences without associated host HLA information. The red line displays the true underlying HLA associated selection of the parametric bootstrap. The blue line displays the median estimate. Grey and white bands display the 5% – 95% and 25% – 75% credible intervals. In the third row, we display the coverage for the 95% and 50% credible intervals in blue and black respectively. The solid lines are using the gold standard reference set, and the dotted lines are using reference data from all public databases. In each of the fourth rows we display the average RMSE at each site for different $\mathbf{D}_{\mathrm{B}}$ as defined in the key. Source data are provided as a Source Data file.

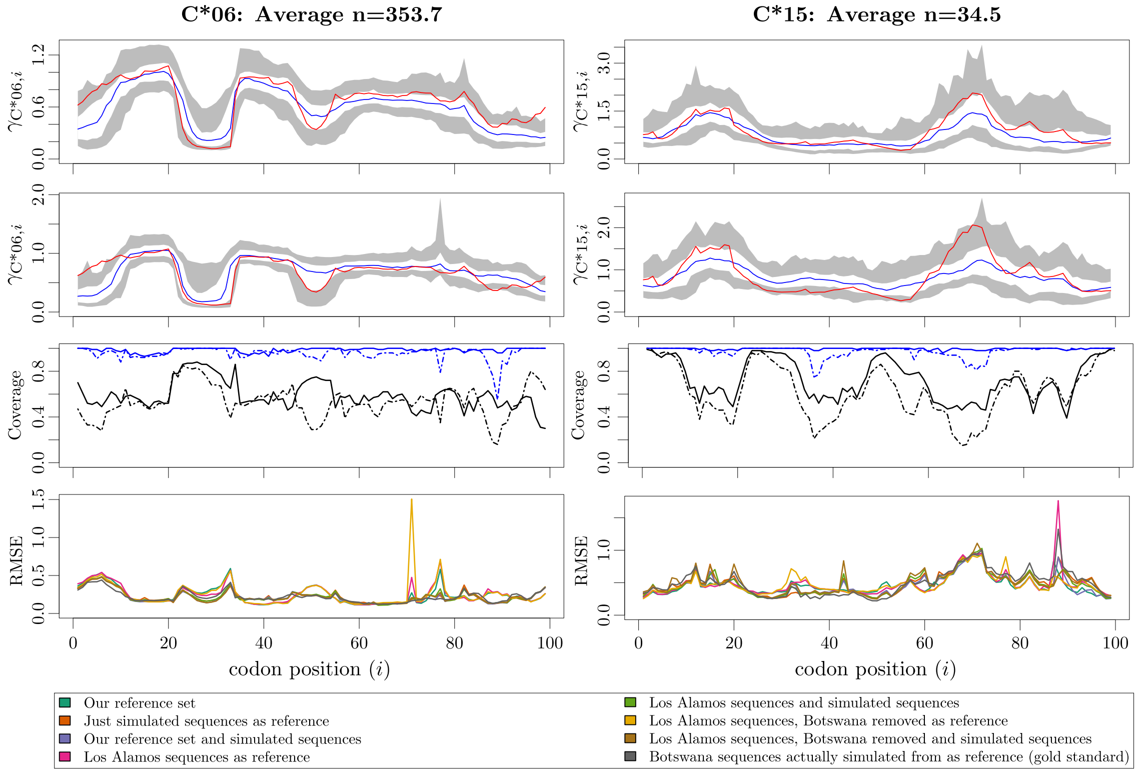

Figure 11: Simulation Study 3: example results for HLA-C alleles. We show results for one rare and one common allele for each of HLA-C. The first panel for each shows the gold standard - if we knew the sequences copied from to create $\mathbf{D}$ and included them in $\mathbf{D}_{\mathrm{B}}$. The second shows the results if we let $\mathbf{D}_{\mathrm{B}}$ be all sequences without associated host HLA information. The red line displays the true underlying HLA associated selection of the parametric bootstrap. The blue line displays the median estimate. Grey and white bands display the 5% – 95% and 25% – 75% credible intervals. In the third row, we display the coverage for the 95% and 50% credible intervals in blue and black respectively. The solid lines are using the gold standard reference set, and the dotted lines are using reference data from all public databases. In each of the fourth rows we display the average RMSE at each site for different $\mathbf{D}_{\mathrm{B}}$ as defined in the key. Source data are provided as a Source Data file.

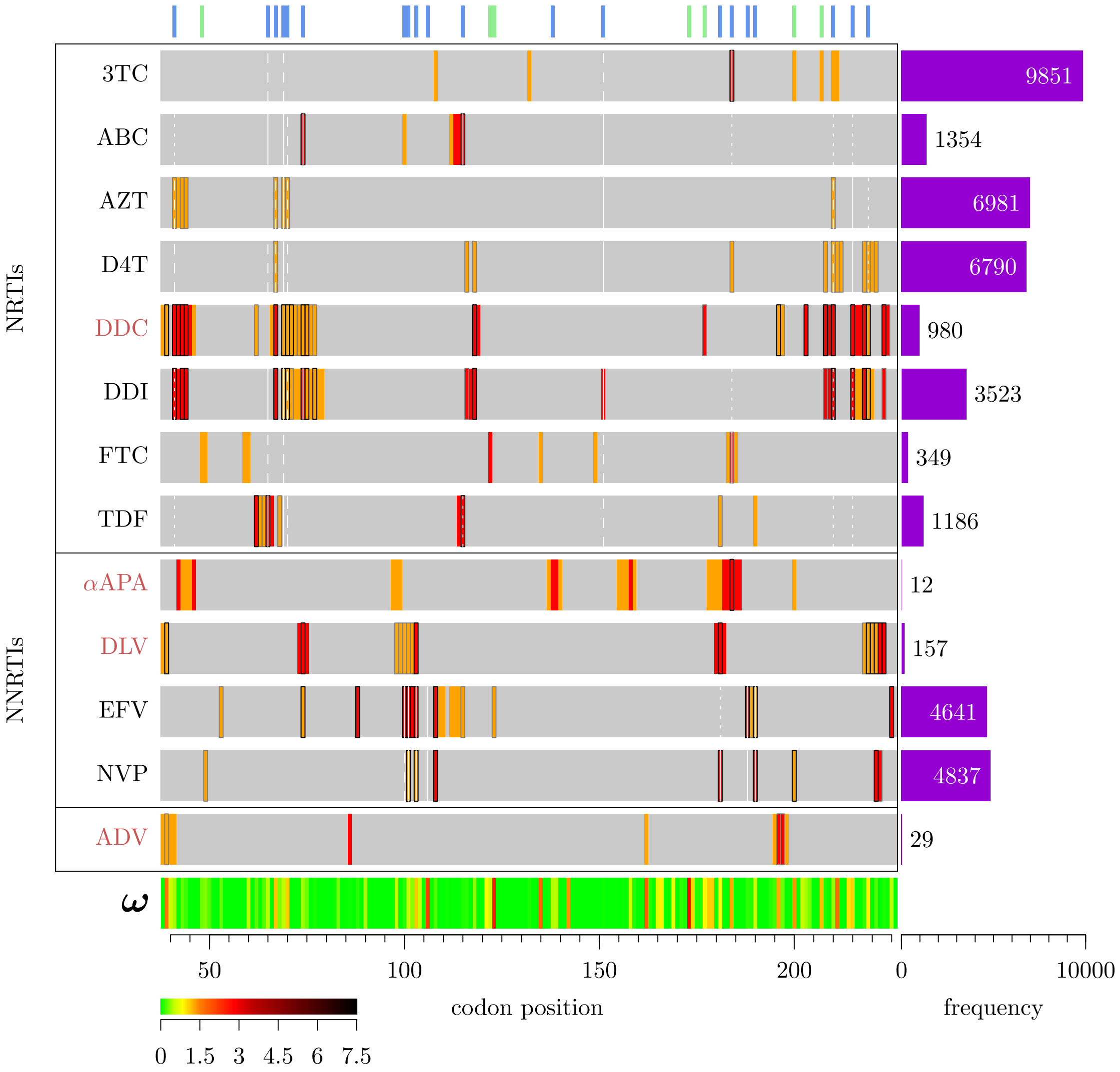

Figure 12: Drug associated selection analysis: Reverse transcriptase results summary. Grey bars highlight the region analysed. Codon position is measured relative to the HXB2 from the start of *reverse transcriptase*. Rows summarise drug-associated selection – drugs are shown to the left. Purple bars show the number of individuals prescribed the drug at viral sampling. Red and orange indicate our median estimate is > 2 and > 1.5 respectively. Sites are outlined in Black and grey if in addition the 2.5% and 10% quantile is > 1 respectively. Green and blue lines at the top of the plots highlight differences between subtype B/C at the amino-acid level, and sites of DRMs [22] respectively. Classes of DRM are displayed: solid white lines tag sites of DRMs which confer the highest levels of resistance for that drug, dashed white lines tag major DRMs, and dotted white lines tag DRMs when combined with other mutations [22]. Drugs highlighted red do not have major DRMs in the Stanford drug resistance database. The different classes of reverse transcriptase inhibitor are separated by black boxes and labelled. ADV is the only nucleotide reverse transcriptase inhibitor (ntRTI). Median ω is displayed at the foot of the figure, according to the colourbar.
Key: 3TC = Lamivudine; ABC = Abacavir; AZT = Zidovudine; D4T = Stavudine; DDC = Zalcitabine; DDI = Didanosine; FTC = Emtricitabine; TDF = Tenofovir; αAPA = Alpha-anilinophenylacetamide; DLV = Delavirdine; EFV = Efavirenz; NVP = Nevirapine; ADV = Adefovir. Source data are provided as a Source Data file.

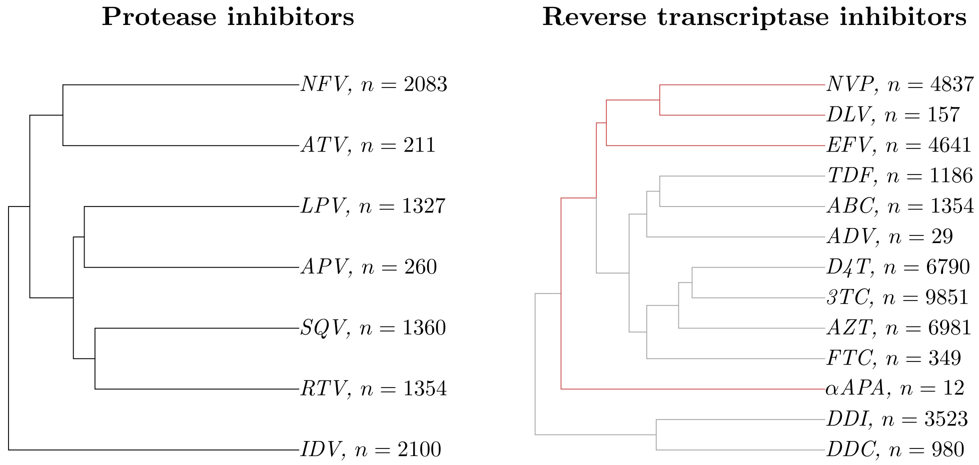

Figure 13: Hierarchical clustering of drug selection profiles. In the reverse transcriptase dendrogram, nNRTIs are highlighted in red, and NRTIs are shown in black. The number of sampled individuals taking each drug as part of their treatment regime are shown to the right of the drug abbreviation. Drug abbreviations are as in Figure 3 and 12 for protease and reverse transcriptase inhibitors respectively. Methods are provided in the Supplementary Methods; Dendrograms of selection profiles and comparing topologies. Source data are provided as a Source Data file.

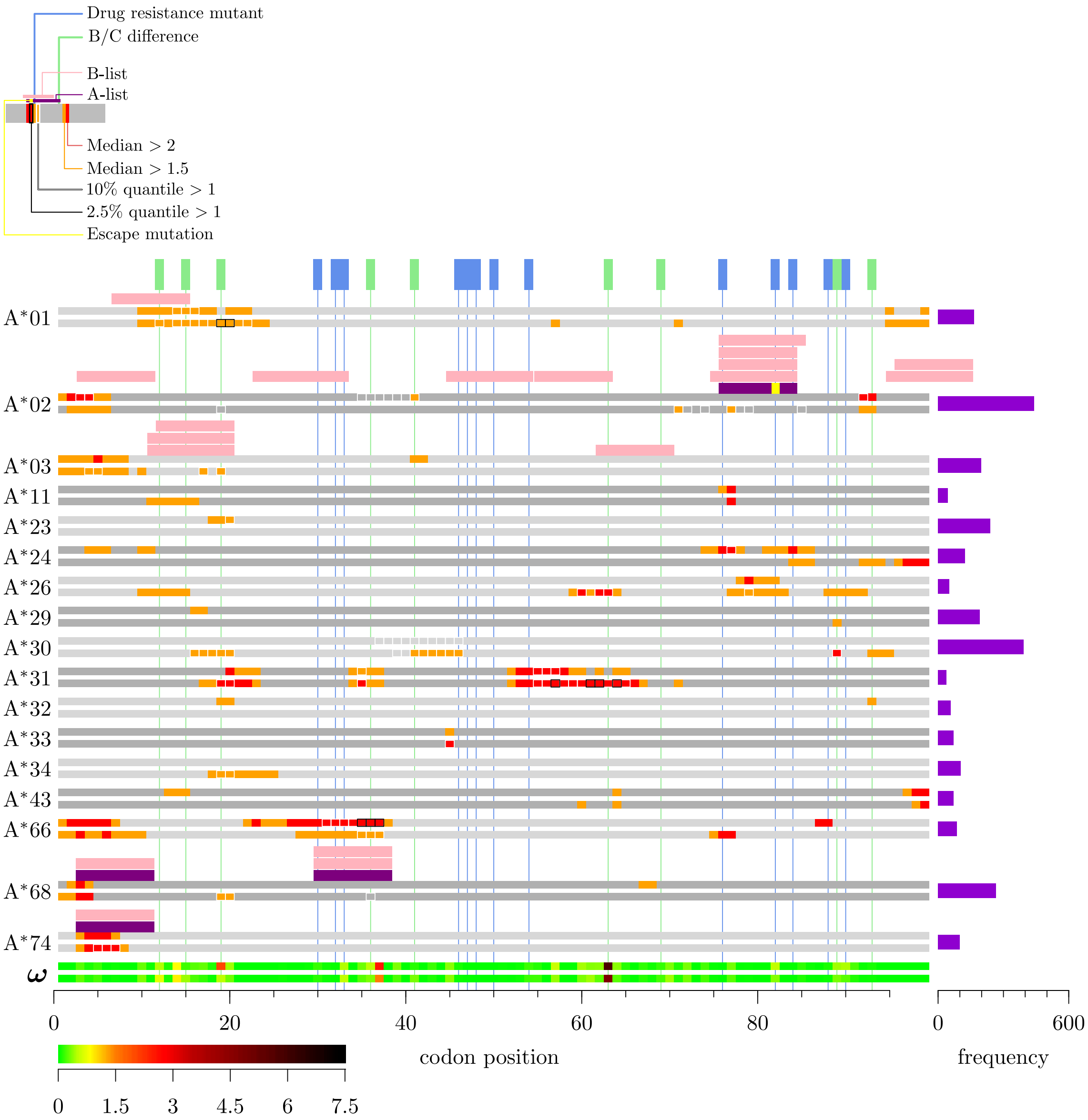

Figure 14: Summary of HLA-A associated selection in protease. We display a summary of all the results of each of our MCMC runs applied to protease. Inference results on the grey horizontal strips are coloured as shown in the key. Two grey strips per HLA show selection away from $\mathscr{C}$ = B and $\mathscr{C}$ = C respectively. Bar plots to the right show HLA frequencies. The two lowest strips summarise median $\boldsymbol{\omega}$ according to the colour bar. Blue vertical lines denote drug resistance sites. Green vertical lines denote sites at which there is an amino acid difference between the subtype B and subtype C consensus viral sequence. Colouring of epitopes plotted above the grey lines refers to the A-list [45] and B-list [46] which are coloured purple and pink respectively. In addition, we colour sites of known escape variants within the A-list epitopes in yellow.

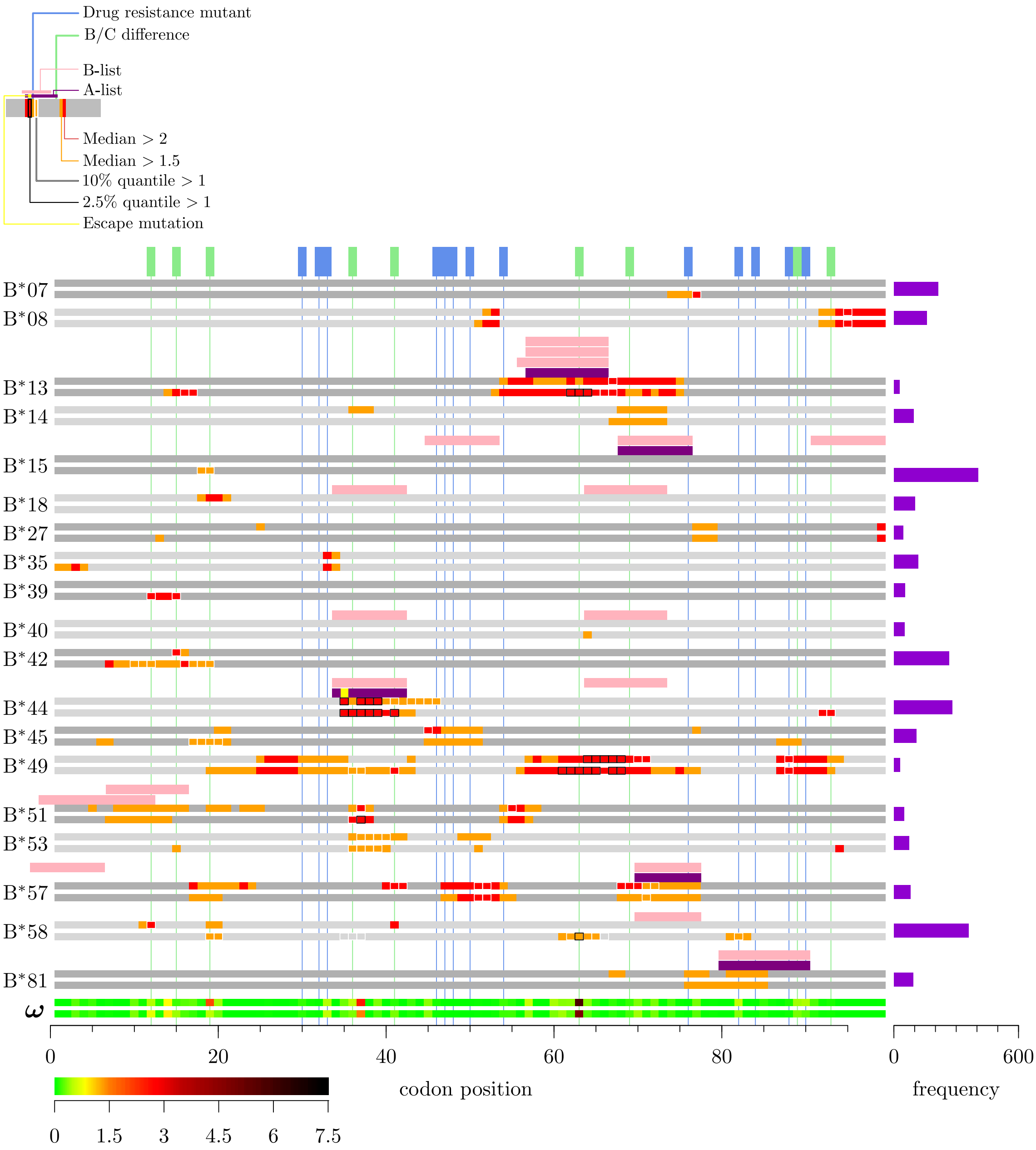

Figure 15: Summary of HLA-B associated selection in protease. We display a summary of all the results of each of our MCMC runs applied to protease. Inference results on the grey horizontal strips are coloured as shown in the key. Two grey strips per HLA show selection away from $\mathscr{C}$ = B and $\mathscr{C}$ = C respectively. Bar plots to the right show HLA frequencies. The two lowest strips summarise median $\boldsymbol{\omega}$ according to the colour bar. Blue vertical lines denote drug resistance sites. Green vertical lines denote sites at which there is an amino acid difference between the subtype B and subtype C consensus viral sequence. Colouring of epitopes plotted above the grey lines refers to the A-list [45] and B-list [46] which are coloured purple and pink respectively. In addition, we colour sites of known escape variants within the A-list epitopes in yellow. Source Data are provided as a Source Data file.

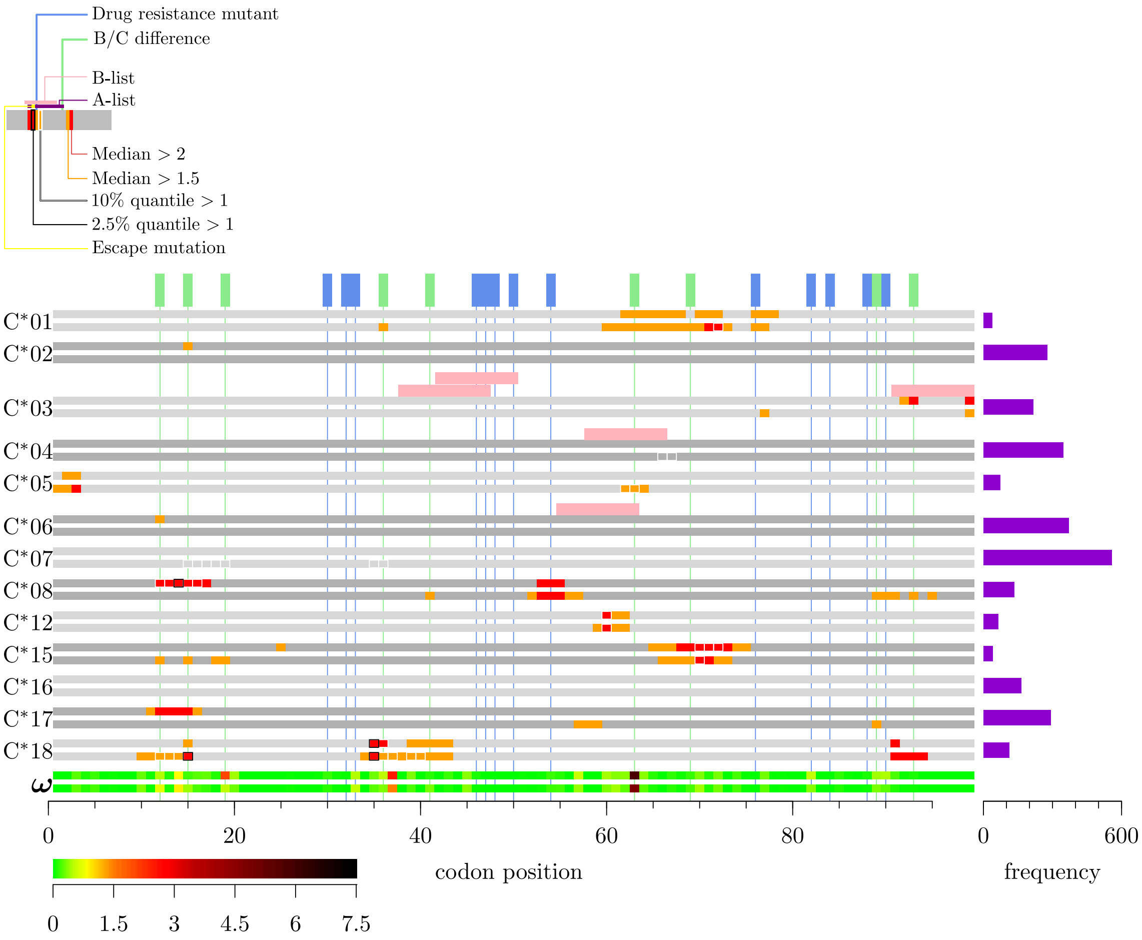

Figure 16: Summary of HLA-C associated selection in protease. We display a summary of all the results of each of our MCMC runs applied to protease. Inference results on the grey horizontal strips are coloured as shown in the key. Two grey strips per HLA show selection away from $\mathscr{C} = \mathrm{B}$ and $\mathscr{C} = \mathrm{C}$ respectively. Bar plots to the right show HLA frequencies. The two lowest strips summarise median $\boldsymbol{\omega}$ according to the colour bar. Blue vertical lines denote drug resistance sites. Green vertical lines denote sites at which there is an amino acid difference between the subtype B and subtype C consensus viral sequence. Colouring of epitopes plotted above the grey lines refers to the A-list [45] and B-list [46] which are coloured purple and pink respectively. In addition, we colour sites of known escape variants within the A-list epitopes in yellow. Source data are provided as a Source Data file.

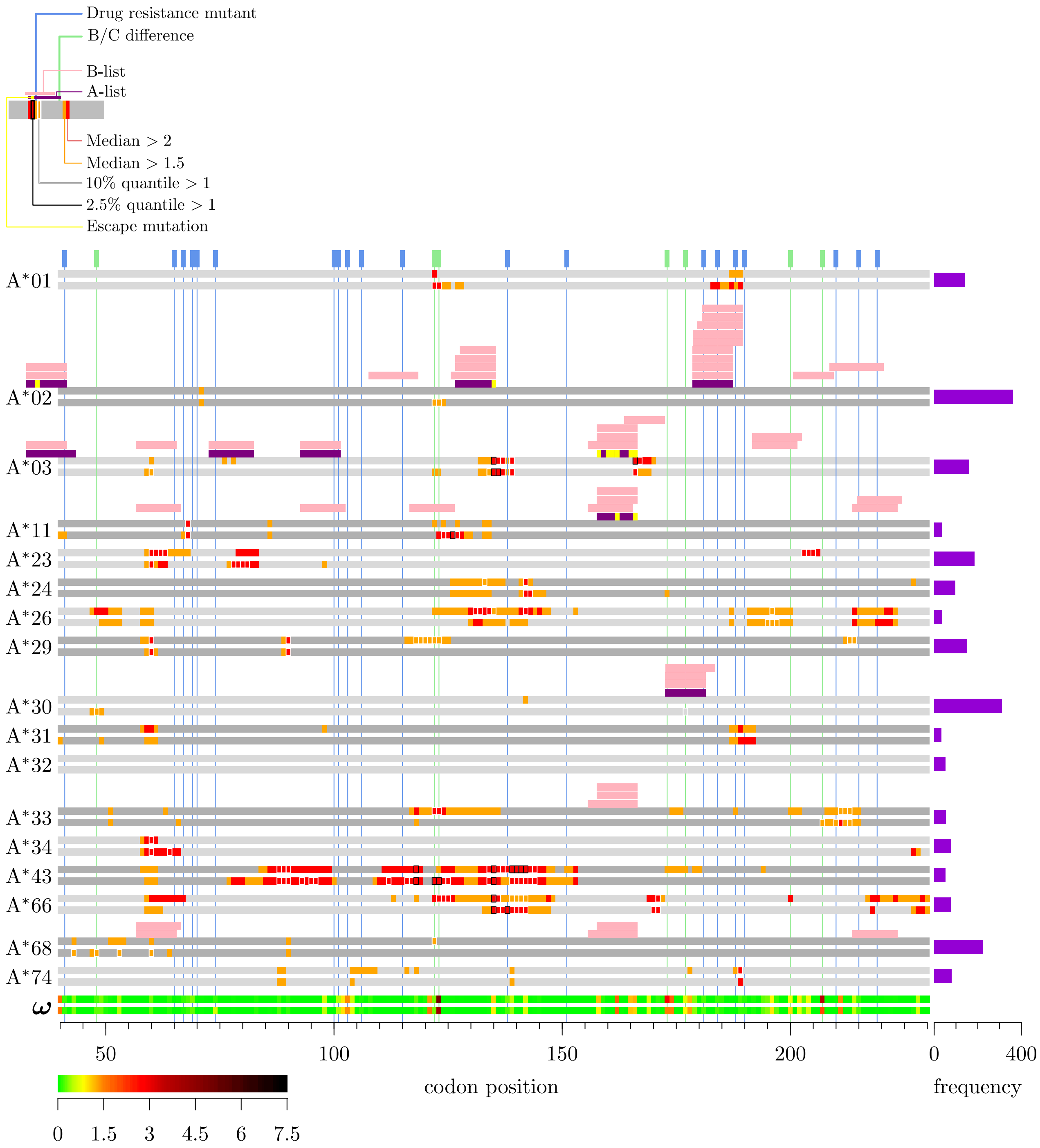

Figure 17: Summary of HLA-A associated selection in reverse transcriptase. We display a summary of all the results of each of our MCMC runs applied to protease. Inference results on the grey horizontal strips are coloured as shown in the key. Two grey strips per HLA show selection away from $\mathscr{C} = \mathrm{B}$ and $\mathscr{C} = \mathrm{C}$ respectively. Bar plots to the right show HLA frequencies. The two lowest strips summarise median $\boldsymbol{\omega}$ according to the colour bar. Blue vertical lines denote drug resistance sites. Green vertical lines denote sites at which there is an amino acid difference between the subtype B and subtype C consensus viral sequence. Colouring of epitopes plotted above the grey lines refers to the A-list [45] and B-list [46] which are coloured purple and pink respectively. In addition, we colour sites of known escape variants within the A-list epitopes in yellow. Source data are provided as a Source Data file.

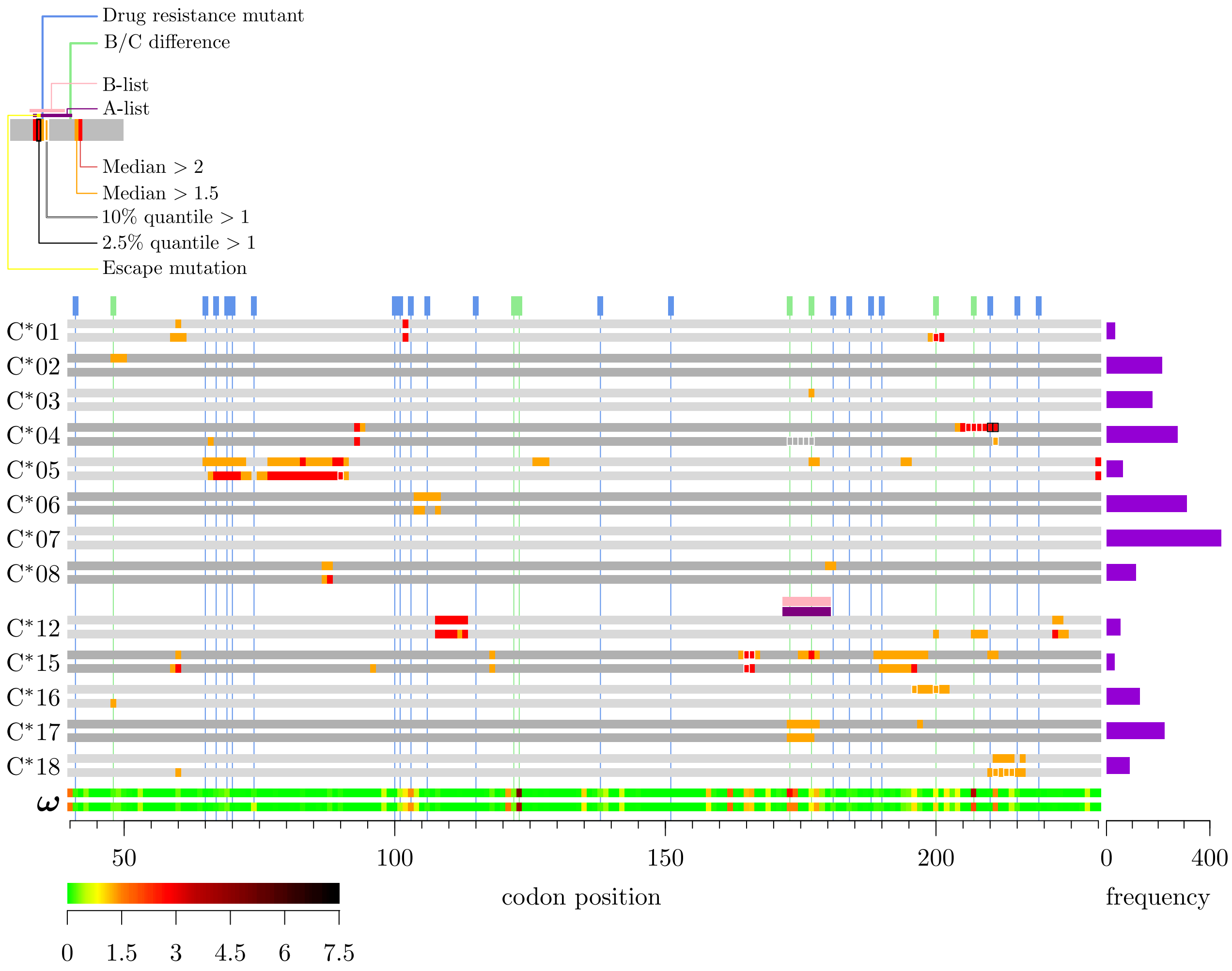

Figure 18: Summary of HLA-C associated selection in reverse transcriptase. We display a summary of all the results of each of our MCMC runs applied to protease. Inference results on the grey horizontal strips are coloured as shown in the key. Two grey strips per HLA show selection away from $\mathscr{C}$ = B and $\mathscr{C}$ = C respectively. Bar plots to the right show HLA frequencies. The two lowest strips summarise median $\boldsymbol{\omega}$ according to the colour bar. Blue vertical lines denote drug resistance sites. Green vertical lines denote sites at which there is an amino acid difference between the subtype B and subtype C consensus viral sequence. Colouring of epitopes plotted above the grey lines refers to the A-list [45] and B-list [46] which are coloured purple and pink respectively. In addition, we colour sites of known escape variants within the A-list epitopes in yellow. Source data are provided as a Source Data file.

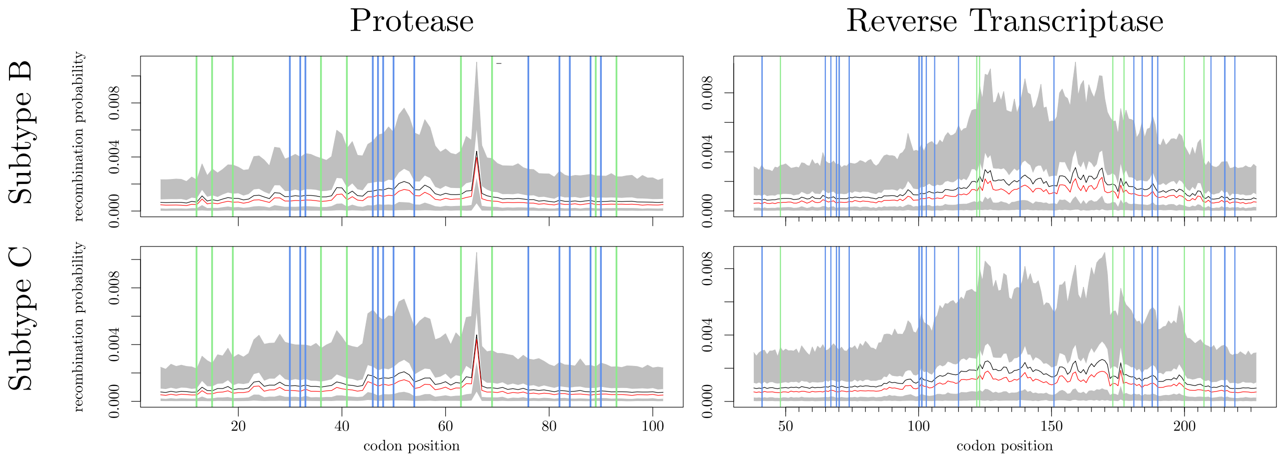

Figure 19: Estimated recombination probabilities between sites across protease and reverse transcriptase. The mean estimate is plotted in black, the median is plotted in red. The white band denotes the 50% credible interval within the 95% credible interval denoted by the grey band. Blue vertical lines denote drug resistance sites. Green vertical lines denote sites at which there is an amino acid difference between the subtype B and subtype C consensus viral sequence. Source data are provided as a Source Data file.

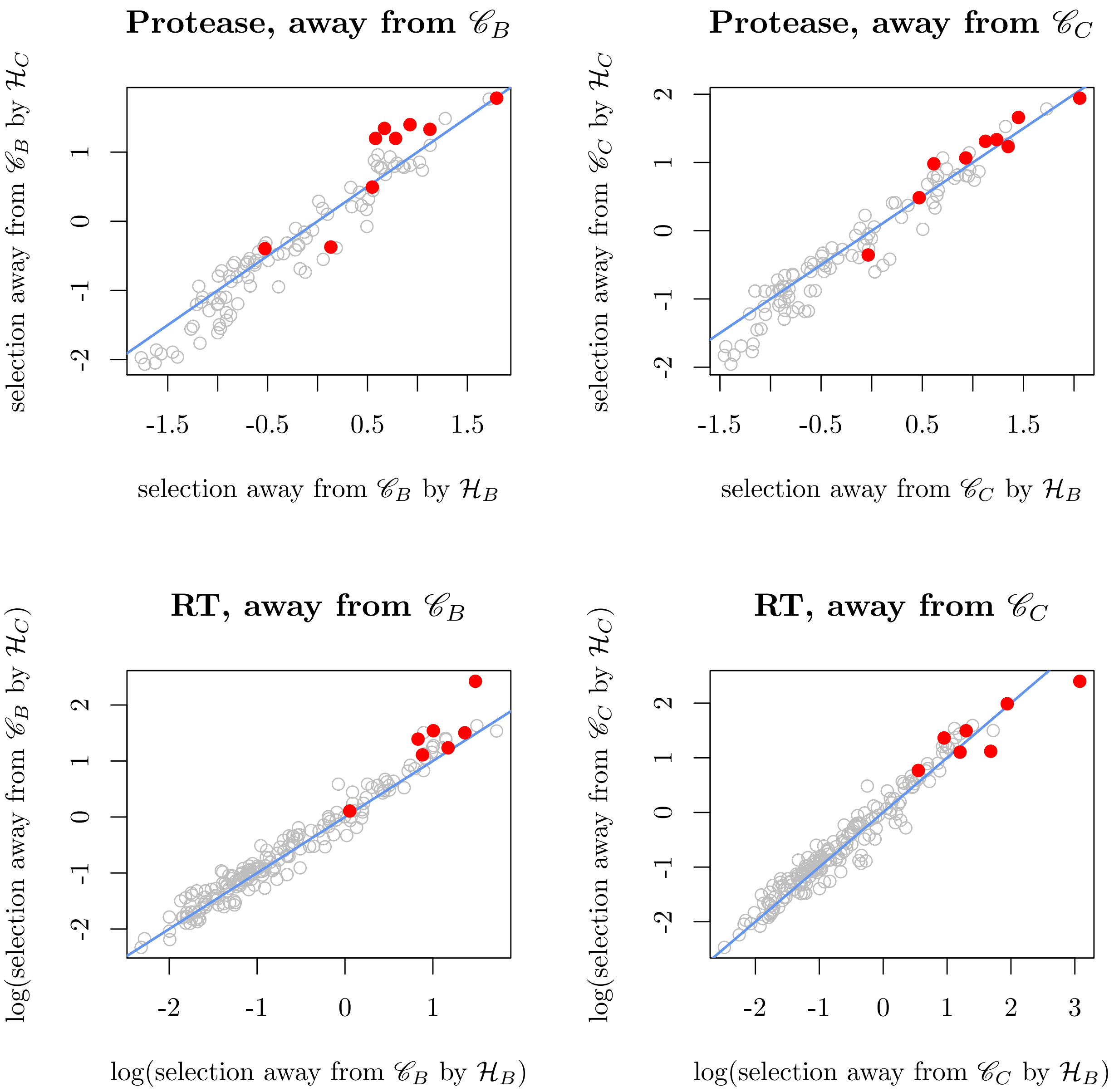

Figure 20: The impact of HLA alleles on differentiation between HIV-1 subtypes. Let $\mathscr{C}_B$ and $\mathscr{C}_B$ be the subtype B and subtype C viral sequence consensus respectively. Let $\mathcal{H}_B$ and $\mathcal{H}_C$ be the distribution of HLA types in individuals harbouring subtype B and subtype C viruses as defined by RIP [47] in the query data set. The panels display comparisons of HLA-associated selection pressure away from $\mathscr{C}_B$ and $\mathscr{C}_C$ by $\mathcal{H}_B$ and $\mathcal{H}_C$. The first row shows results for protease, and the second row shows results for reverse transcriptase. Sites that distinguish subtype B and subtype C at the amino acid level are highlighted in red. Source data are provided as a Source Data file.

Supplementary Tables

Parameter	Prior
μ	$Exp(0.01)$
ν_i	$Exp(m)$,
	where m is chosen such that $E(r_i) = 0.01$
ω_i	$Exp(0.5)$
ζ_i	$\log \mathcal{N}(0, 4)$
$\gamma_{h,i}$	$\log \mathcal{N}(0, 4)$
window expansion	$Geom(0.5)$
number of HLA selection windows	$Bin(l, 0.2)$
number of reversion windows	$Bin(l, 0.3)$

Table 1: Priors on parameters of the model. l is the length of analysed sequences in codons, i and h enumerate codon site and HLA type respectively.

Protein	Drug	Known DRM	Selected by cocktail involving drug	Selected by drug class	Selected by different drug in same drug class	Not known to be selected by any drug
Protease	APV			11	34, 55.	
	IDV	10, 24, 71, 73, 85, 93.	66, 95.	92	41, 55.	72
	LPV	53, 55, 91.				
	NFV	77		92	13, 41, 93.	
	SQV	71, 73.	53	74, 83, 85.		
Reverse Transcriptase	DDI	67		118, 218.		48, 49.
	TDF			62		
	EFV	108, 225.				88, 102.
	NVP	108, 221.				

Table 2: The collection of 'false positive' associations are interrogated. We find that many of these associations, though not in the collection of major drug resistance mutations [22], are documented to be under selection.

Protein	Drug	2-step mutation	Indel	*In vitro* data only	No documented evidence for selection	False negative
Protease	ATV			84V, 88S.		
	NFV			82AFTS, 84A.	48VM, 54VTALM.	
Reverse Transcriptase	ABC	151M	69Ins	65R		
	AZT	151M, 215FY.				
	D4T	151M, 215FY.	69Ins			
	TDF	151M		65R		
	EFV	106M	69Ins			106A
	NVP	188L, 106M.				106A, 188CH.

Table 3: The collection of apparent false negative associations are interrogated. We can explain most false negatives - either due to our modelling regime, or lack of evidence for a true selective effect.

HLA	$\mathscr{C} = B$	$\mathscr{C} = C$
A*01	14,15,16	12,14,15,16,17,18,19,20,21,22
A*02	3,4,35,36,37,38,39,40,41,92	19,71,72,74,77,78,79,85
A*03		4,5,17,19
A*23	20	
A*24	77	
A*26		60,61,62,63,79
A*30	37,38,39,40,41,42,43,44,45,46	16,17,18,19,20,39,40,41,42,43,44,45,46,89
A*31	35,55,56,57	19,20,35,55,56,57,58,59,60,61,62,63,64,65
A*33		45
A*34		19,20
A*66	31,32,33,34,35,36,37	35,36,37
A*68		19,20,36
A*74		5,6,7
B*07		77
B*08	95	95
B*13	67	16,17,62,63,64,65,66,67
B*15		18,19
B*39		12,15
B*42	15	10,11,12,16,17,18,19
B*44	35,37,38,39,40,41,43,44,45,46	35,36,37,38,39,40,41,92,93
B*45	45	17,18,19,20
B*49	64,65,66,67,68,70,71,88	36,37,41,61,62,63,64,65,66,67,68,88
B*51	37,55	36,37
B*53	37,38,39,40	36,37,38,39
B*57	41,42,51,52,68,69,70,71,72	51,52,71
B*58	12	19,20,35,36,37,62,63,64,65,66,82
C*01		72
C*04		66,67
C*05		62,63
C*07		15,16,17,18,19,35,36
C*08	12,13,14,15,16	
C*12	60	60
C*15	70,71,72	70
C*18	35,36	12,13,14,15,35,36,37,39,40

Table 4: The collection of top-tier and second-tier sites in protease. Top-tier sites - median $\gamma_{h,i} > 2$ and with the lower 2.5% quantile > 1 are underlined. The remainder are second-tier sites, with the lower 10% quantile > 1. Source data are provided as a Source Data file.

HLA	$\mathscr{C} = B$	$\mathscr{C} = C$
A*01		122,123
A*02		122,123
A*03	135,136,137,138,139,166,167	60,134,135,136,137,138,139,166
A*11	68	68,124,125,126,127
A*23	60,61,62,63,203,204,205	60,78,79,80,81
A*24	133,142	142,143
A*26	131,132,134,135,142,196	195,196,197
A*29	60,90,118,119,120,121,122,123,213,214	60,90
A*30		48,177
A*33	122,123,211,212,213	207,210,211,212,213
A*34	60	60,64
A*43	88,89,90,118,134,135,136,137,138,139,140, 141,142,143,144	88,89,90,93,95,96,112,116,117,118,122,123, 124,125,134,135,139,140,141,142,143,144
A*66	123,124,125,135,136,139,140,141,142,171	135,136,137,138,139,140,141,142,170,171
A*68	122	43,48,53,60
A*74	189	
B*07	158,159,160,161,162,163,164,165	158,159,160,161,162,163,164,165
B*08	88,89,90,91,211,212,230	89,90,91,230
B*14	43,60	60
B*15	60	60
B*18	144	138,144,145,177,178
B*27	142	127,128,142
B*35	122	122,123
B*39	83	
B*44		125,204,207
B*45	123,135,196,211,212,213,214	67,68,69,135,196,197,202,203,204,205,206, 207,208,209,210,211,212,213,214,215
B*51	121,122,123,134,135,196	135,173,174,175,196
B*53		123
B*57	118,211,212,213,214,215	118,122,211,212,213,214,215
B*58	122,123	173,174,175,176,177
B*81	118,122,123,158,214	118,158,214
C*01		200
C*04	206,207,208,209,210,211	173,174,175,176,177,211
C*05		90
C*15	165,166	165
C*16	196,200	
C*18		211,213,214

Table 5: The collection of top-tier and second tier sites in reverse-transcriptase. Top-tier sites - median $\gamma_{h,i} > 2$ and with the lower 2.5% quantile > 1 are underlined. The remainder are second-tier sites, with the lower 10% quantile > 1. Source data are provided as a Source Data file.

HLA	Both	Top-tier	Carlson $q < 0.2$ [48]
B*07			36
B*08			57
B*13		62,63,64	
B*15			19,71,74
B*27			19
B*42			15
B*44	35,36,37,41	38,39	82
B*49		61,62,63,64,65,67,68	
B*51		37	
B*58			37,63

Table 6: Overlap between studies. We compare our top-tier sites in Protease to those found in Carlson *et al.* [48] with $q < 0.2$. The both column denotes sites that were found in both studies. Sites that lie within known A or B-list epitopes are underlined. Source data are provided as a Source Data file.

HLA	Both	Top-tier	Carlson $q < 0.2$ [48]
B*07	162,165	159,163,164	
B*15			48,174
B*18	138		177
B*27		142	
B*35	122,123		121,173,196
B*42			162
B*44	204,207		
B*45		196,203,204,207,211	
B*51	135	173	
B*53		123	
B*81		118	

Table 7: Overlap between studies. We compare our top-tier sites in Reverse Transcriptase to those found in Carlson *et al.* [48] with $q < 0.2$. The both column denotes sites that were found in both studies. Sites that lie within known A or B-list epitopes [49] are underlined. Source data are provided as a Source Data file.

Consensus	Region	
	Protease	Reverse Transcriptase
	Selection due to $\mathcal{H}_C > \mathcal{H}_B$	
$\mathscr{C}_B$	$p = 2.8 \times 10^{-5}$	$p = 2.0 \times 10^{-6}$
	Selection due to $\mathcal{H}_B > \mathcal{H}_C$	
$\mathscr{C}_C$	$p = 0.70$	$p = 0.0058$

Table 8: Table of p-values for differences between $\mathcal{H}_B$ and $\mathcal{H}_C$ associated selection at sites that distinguish subtype B and subtype C at the amino acid level in protease and reverse transcriptase. Source data are provided as a Source Data file.

Reference set	Size	RMSE	Coverage 95%	Coverage 50%
Gold standard	343	0.766	97.7%	57.4%
All public databases	162,901	0.767	90.7%	45.7%
LOO: Simulated sequences	1,500	0.796	96.2%	53.2%
LOO: All public databases + simulated sequences	164,401	0.783	96.3%	52.9%
Los Alamos database	58,042	0.791	90.3%	45.1%
Los Alamos database – Botswana	57,969	0.788	90.7%	45.9%
LOO: Los Alamos database	59,452	0.786	96.1%	52.7%
LOO: Los Alamos database – Botswana	59,469	0.789	96.2%	52.9%

Table 9: Summary of reference data sets for simulation study 3. Source data are provided as a Source Data file.

Supplementary References

1. Li, N. & Stephens, M. Modeling Linkage Disequilibrium and Identifying Recombination Hotspots Using Single-Nucleotide Polymorphism Data. *Genetics* **165,** 2213–2233. ISSN: 1943-2631 (Dec. 2003).
2. Nielsen, R. & Yang, Z. Likelihood Models for Detecting Positively Selected Amino Acid Sites and Applications to the HIV-1 Envelope Gene. *Genetics* **148,** 929–936. ISSN: 1943-2631 (Mar. 1998).
3. Rabiner, L. R. *A tutorial on hidden Markov models and selected applications in speech recognition* in *PROCEEDINGS OF THE IEEE* **77** (1989), 257–286. `http://citeseerx.ist.psu.edu/viewdoc/summary?doi=10.1.1.131.2084`.
4. Wilson, D. J. & McVean, G. Estimating diversifying selection and functional constraint in the presence of recombination. *Genetics* **172,** 1411–1425. ISSN: 0016-6731 (Mar. 2006).
5. Crawford, D. C. *et al.* Evidence for substantial fine-scale variation in recombination rates across the human genome. *Nature Genetics* **36,** 700–706. ISSN: 1061-4036 (June 2004).
6. Moore, C. B. *et al.* Evidence of HIV-1 adaptation to HLA-restricted immune responses at a population level. *Science (New York, N.Y.)* **296,** 1439–1443. ISSN: 1095-9203 (May 2002).
7. Bhattacharya, T. *et al.* Founder Effects in the Assessment of HIV Polymorphisms and HLA Allele Associations. *Science* **315,** 1583–1586 (Mar. 2007).
8. Fryer, H. R. *et al.* Cytotoxic T-lymphocyte escape mutations identified by HLA association favor those which escape and revert rapidly. *Journal of virology* **86,** 8568–8580. ISSN: 1098-5514 (Aug. 2012).
9. Carlson, J. M. *et al.* Phylogenetic dependency networks: inferring patterns of CTL escape and codon covariation in HIV-1 Gag. *PLoS computational biology* **4,** e1000225+. ISSN: 1553-7358 (Nov. 2008).
10. Carlson, J. M. *et al.* Widespread Impact of HLA Restriction on Immune Control and Escape Pathways of HIV-1. *Journal of Virology* **86,** 5230–5243. ISSN: 1098-5514 (May 2012).
11. Stamatakis, A., Ludwig, T. & Meier, H. RAxML-III: a fast program for maximum likelihood-based inference of large phylogenetic trees. *Bioinformatics (Oxford, England)* **21,** 456–463. ISSN: 1367-4803 (Feb. 2005).
12. Fryer, H. R. *et al.* Modelling the Evolution and Spread of HIV Immune Escape Mutants. *PLoS Pathog* **6,** e1001196+. ISSN: 1553-7374 (Nov. 2010).
13. Felsenstein, J. Evolutionary trees from DNA sequences: a maximum likelihood approach. *Journal of molecular evolution* **17,** 368–376. ISSN: 0022-2844 (1981).
14. Carlson, J., Kadie, C., Mallal, S. & Heckerman, D. Leveraging Hierarchical Population Structure in Discrete Association Studies. *PLoS ONE* **2,** e591+ (July 2007).
15. Nash, J. C. & Varadhan, R. Unifying Optimization Algorithms to Aid Software System Users: optimx for R. *Journal of Statistical Software* **43,** 1–14 (Aug. 2011).

16. Nelder, J. A. & Mead, R. A Simplex Method for Function Minimization. *The Computer Journal* **7,** 308–313. ISSN: 1460-2067 (Jan. 1965).
17. Byrd, R. H., Lu, P., Nocedal, J. & Zhu, C. A Limited Memory Algorithm for Bound Constrained Optimization. *SIAM J. Sci. Comput.* **16,** 1190–1208. ISSN: 1064-8275 (Sept. 1995).
18. Bruun Nielsen, H., Universitet, D. T. & for Matematisk Modellering, I. *Ucminf - an algorithm for unconstrained, nonlinear optimization* `http://www.worldcat.org/oclc/461923122` (IMM, Department of Mathematical Modelling, Technical University of Denmark, 2000).
19. Kelley, C. T. *Iterative Methods for Optimization (Frontiers in Applied Mathematics)* 1st ed. ISBN: 0898714338. `http://www.worldcat.org/isbn/0898714338` (Society for Industrial and Applied Mathematics, Jan. 1987).
20. Powell, M. J. D. in *Large-Scale Nonlinear Optimization* (eds Di Pillo, G. & Roma, M.) 255–297 (Springer US, Boston, 2006). ISBN: 0-387-30063-5. doi:`10.1007/0-387-30065-1\_16`. `http://dx.doi.org/10.1007/0-387-30065-1\_16`.
21. Powell, M. J. D. The BOBYQA algorithm for bound constrained optimization without derivatives (Aug. 2009).
22. *Stanford drug resistance database* `http://hivdb.stanford.edu/`.
23. Fagard, C. *et al.* A prospective trial of structured treatment interruptions in human immunodeficiency virus infection. *Archives of internal medicine* **163,** 1220–1226. ISSN: 0003-9926 (May 2003).
24. Frater, A. J. *et al.* Effective T-Cell Responses Select Human Immunodeficiency Virus Mutants and Slow Disease Progression. *Journal of Virology* **81,** 6742–6751. ISSN: 1098-5514 (June 2007).
25. Leslie, A. *et al.* Additive Contribution of HLA Class I Alleles in the Immune Control of HIV-1 Infection. *Journal of Virology* **84,** 9879–9888. ISSN: 1098-5514 (Oct. 2010).
26. Matthews, P. C. *et al.* Central Role of Reverting Mutations in HLA Associations with Human Immunodeficiency Virus Set Point. *Journal of Virology* **82,** 8548–8559. ISSN: 1098-5514 (Sept. 2008).
27. Shapiro, R. L. *et al.* Antiretroviral Regimens in Pregnancy and Breast-Feeding in Botswana. *N Engl J Med* **362,** 2282–2294 (June 2010).
28. Huang, K.-H.G. H. *et al.* Prevalence of HIV type-1 drug-associated mutations in pre-therapy patients in the Free State, South Africa. *Antiviral therapy* **14,** 975–984. ISSN: 1359-6535 (2009).
29. Huang, K.-H. G. *et al.* Progression to AIDS in South Africa Is Associated with both Reverting and Compensatory Viral Mutations. *PLoS ONE* **6,** e19018+ (Apr. 2011).
30. Short-Course Antiretroviral Therapy in Primary HIV Infection. *N Engl J Med* **368,** 207–217 (Jan. 2013).
31. *Los Alamos HIV sequence database* `http://www.hiv.lanl.gov/`.
32. Edgar, R. C. MUSCLE: a multiple sequence alignment method with reduced time and space complexity. *BMC bioinformatics* **5,** 113+. ISSN: 1471-2105 (Aug. 2004).
33. Robinson, J. *et al.* The IMGT/HLA database. *Nucleic Acids Research* **41,** D1222–D1227. ISSN: 1362-4962 (Jan. 2013).
34. Boutwell, C. L. & Essex, M. Identification of HLA class I-associated amino acid polymorphisms in the HIV-1C proteome. *AIDS research and human retroviruses* **23,** 165–174. ISSN: 0889-2229 (Jan. 2007).
35. Brumme, Z. L. *et al.* Marked epitope- and allele-specific differences in rates of mutation in human immunodeficiency type 1 (HIV-1) Gag, Pol, and Nef cytotoxic T-lymphocyte epitopes in acute/early HIV-1 infection. *Journal of virology* **82,** 9216–9227. ISSN: 1098-5514 (Sept. 2008).
36. Hoof, I. *et al.* Interdisciplinary analysis of HIV-specific CD8+ T cell responses against variant epitopes reveals restricted TCR promiscuity. *Journal of immunology (Baltimore, Md. : 1950)* **184,** 5383–5391. ISSN: 1550-6606 (May 2010).
37. Kiepiela, P. *et al.* CD8+ T-cell responses to different HIV proteins have discordant associations with viral load. *Nature medicine* **13,** 46–53. ISSN: 1078-8956 (Jan. 2007).
38. Liu, Y., McNevin, J. P., Holte, S., McElrath, M. J. & Mullins, J. I. Dynamics of viral evolution and CTL responses in HIV-1 infection. *PloS one* **6.** ISSN: 1932-6203. `http://view.ncbi.nlm.nih.gov/pubmed/21283794` (Jan. 2011).

39. Mueller, S. M. *et al.* Dual selection pressure by drugs and HLA class I-restricted immune responses on human immunodeficiency virus type 1 protease. *Journal of virology* **81,** 2887–2898. ISSN: 0022-538X (Mar. 2007).
40. Ferrari, G. *et al.* Relationship between functional profile of HIV-1 specific CD8 T cells and epitope variability with the selection of escape mutants in acute HIV-1 infection. *PLoS pathogens* **7.** ISSN: 1553-7374. doi:10.1371/journal.ppat.1001273. http://dx.doi.org/10.1371/journal.ppat.1001273 (2011).
41. Draenert, R. *et al.* Constraints on HIV-1 evolution and immunodominance revealed in monozygotic adult twins infected with the same virus. *The Journal of experimental medicine* **203,** 529–539. ISSN: 0022-1007 (Mar. 2006).
42. Koibuchi, T. *et al.* Limited sequence evolution within persistently targeted CD8 epitopes in chronic human immunodeficiency virus type 1 infection. *Journal of virology* **79,** 8171–8181. ISSN: 0022-538X (July 2005).
43. Menéndez-Arias, L., Mas, A. & Domingo, E. Cytotoxic T-lymphocyte responses to HIV-1 reverse transcriptase (review). *Viral immunology* **11,** 167–181. ISSN: 0882-8245 (1998).
44. Stratov, I., Dale, C. J., Chea, S., McCluskey, J. & Kent, S. J. Induction of T-cell immunity to antiretroviral drug-resistant human immunodeficiency virus type 1. *Journal of virology* **79,** 7728–7737. ISSN: 0022-538X (June 2005).
45. Llano, A., Williams, A., Olvera, A., Silva-Arrieta, S. & Brander, C. *Best-Characterized HIV-1 CTL Epitopes: The 2013 Update* tech. rep. (2013). http://www.hiv.lanl.gov/content/immunology/pdf/2013/optimal_ctl_article.pdf.
46. *Collection of B-list epitopes* http://www.hiv.lanl.gov/content/immunology/tables/ctl_summary.html.
47. *Recombinant Identification Program* http://www.hiv.lanl.gov/content/sequence/RIP/RIP.html.
48. Carlson, J. M. *et al.* Impact of pre-adapted HIV transmission. *Nature medicine* **22,** 606–613. ISSN: 1546-170X (June 2016).
49. *Los Alamos HIV immunology database* http://www.hiv.lanl.gov/content/immunology.
