# Supplementary material for: Mapping the drivers of within-host pathogen evolution using massive data sets
Source: Nat Commun. 2019 Jul 9;10:3017. doi: 10.1038/s41467-019-10724-w (PMC6616926; doi:10.1038/s41467-019-10724-w)
Supplement: Supplementary file 3 — Reporting Summary [file 41467_2019_10724_MOESM3_ESM.pdf]

nature research

Corresponding author(s): Dr Duncan Palmer

Last updated by author(s): Apr 30, 2019

Reporting Summary

Nature Research wishes to improve the reproducibility of the work that we publish. This form provides structure for consistency and transparency in reporting. For further information on Nature Research policies, see Authors & Referees and the Editorial Policy Checklist.

Statistics

For all statistical analyses, confirm that the following items are present in the figure legend, table legend, main text, or Methods section.

n/a	Confirmed	
☒	☐	The exact sample size (*n*) for each experimental group/condition, given as a discrete number and unit of measurement
☒	☐	A statement on whether measurements were taken from distinct samples or whether the same sample was measured repeatedly
☐	☒	The statistical test(s) used AND whether they are one- or two-sided<br>*Only common tests should be described solely by name; describe more complex techniques in the Methods section.*
☒	☐	A description of all covariates tested
☐	☒	A description of any assumptions or corrections, such as tests of normality and adjustment for multiple comparisons
☐	☒	A full description of the statistical parameters including central tendency (e.g. means) or other basic estimates (e.g. regression coefficient) AND variation (e.g. standard deviation) or associated estimates of uncertainty (e.g. confidence intervals)
☒	☐	For null hypothesis testing, the test statistic (e.g. *F*, *t*, *r*) with confidence intervals, effect sizes, degrees of freedom and *P* value noted<br>*Give P values as exact values whenever suitable.*
☐	☒	For Bayesian analysis, information on the choice of priors and Markov chain Monte Carlo settings
☒	☐	For hierarchical and complex designs, identification of the appropriate level for tests and full reporting of outcomes
☒	☐	Estimates of effect sizes (e.g. Cohen's *d*, Pearson's *r*), indicating how they were calculated

Our web collection on statistics for biologists contains articles on many of the points above.

Software and code

Policy information about availability of computer code

Data collection	no software was used
Data analysis	data analysed using custom code, available at github.com/astheeggeggs/mcqueen and RAxML 8.2.

For manuscripts utilizing custom algorithms or software that are central to the research but not yet described in published literature, software must be made available to editors/reviewers. We strongly encourage code deposition in a community repository (e.g. GitHub). See the Nature Research guidelines for submitting code & software for further information.

Data

Policy information about availability of data

All manuscripts must include a data availability statement. This statement should provide the following information, where applicable:

- Accession codes, unique identifiers, or web links for publicly available datasets
- A list of figures that have associated raw data
- A description of any restrictions on data availability

Host HLA data used in this study are available from the corresponding author upon reasonable request. Query datasets for drug inference, associated host drug information, and all reference datasets are available in the mcqueen paper repository: www.github.com/astheeggeggs/mcqueen_paper. Source data underlying Figures 2-5, Supplementary Figures 20, Tables 3-6 and Supplementary Tables 4,5,6 and 9 are available in the source data file. Raw MCMC output for simulation studies, HLA, and drug inference are available at www.github.com/astheeggeggs/mcqueen_paper.

Field-specific reporting

Please select the one below that is the best fit for your research. If you are not sure, read the appropriate sections before making your selection.

☒ Life sciences ☐ Behavioural & social sciences ☐ Ecological, evolutionary & environmental sciences

For a reference copy of the document with all sections, see nature.com/documents/nr-reporting-summary-flat.pdf

Life sciences study design

All studies must disclose on these points even when the disclosure is negative.

Sample size	This was an exploratory methods development project that used as much available data as possible. Sample size was based on availability of sequence, drug and host HLA data. Existing research in the literature has lead to reproducible signals of association between host HLA and viral sequence with far smaller sample sizes, we sought to reproduce these and find novel associations.
Data exclusions	We removed sample overlaps from across public HIV sequence databases, and removed repeat entries. After alignment, sites with >5% nucleotide ambiguity were removed, followed by sequences for which there was >5% nucleotide ambiguity.
Replication	We ensured that our method could reproduce signals of drug associated and HLA associated selection in the literature.
Randomization	Allocation of whether patients had HLA typing available was not random, we used all available data in the literature and through collaboration. Controlling for bias in HLA typing was through our modeling framework which controls for correlation structure in viral sequence data.
Blinding	Blinding was not relevant to our study, this was not a case/control or group study design.

Reporting for specific materials, systems and methods

We require information from authors about some types of materials, experimental systems and methods used in many studies. Here, indicate whether each material, system or method listed is relevant to your study. If you are not sure if a list item applies to your research, read the appropriate section before selecting a response.

Materials & experimental systems

n/a	Involved in the study	
☒	☐	Antibodies
☒	☐	Eukaryotic cell lines
☒	☐	Palaeontology
☒	☐	Animals and other organisms
☒	☐	Human research participants
☒	☐	Clinical data

Methods

n/a	Involved in the study	
☒	☐	ChIP-seq
☒	☐	Flow cytometry
☒	☐	MRI-based neuroimaging
